# Longitudinal multimodal imaging-compatible mouse model of triazole-sensitive and -resistant invasive pulmonary aspergillosis

**DOI:** 10.1242/dmm.049165

**Published:** 2022-03-30

**Authors:** Agustin Resendiz-Sharpe, Roberta Peres da Silva, Elena Geib, Lore Vanderbeke, Laura Seldeslachts, Charlien Hupko, Matthias Brock, Katrien Lagrou, Greetje Vande Velde

**Affiliations:** 1 Laboratory of Clinical Microbiology, Department of Microbiology, Immunology and Transplantation, Katholieke Universiteit (KU) Leuven, 3000 Leuven, Belgium; 2 Fungal Biology Group, School of Life Sciences, University of Nottingham, Nottingham NG7 2RD, United Kingdom; 3 Department of Imaging and Pathology, Biomedical MRI unit/MoSAIC, KU Leuven, 3000 Leuven, Belgium; 4 Department of Laboratory Medicine and National Reference Centre for Mycosis, Excellence Centre for Medical Mycology (ECMM), University Hospitals Leuven, 3000 Leuven, Belgium

**Keywords:** Invasive bronchopulmonary aspergillosis, Antifungal resistance, Murine model, Bioluminescence imaging, Micro-CT

## Abstract

Invasive pulmonary aspergillosis (IPA) caused by the mold *Aspergillus fumigatus* is one of the most important life-threatening infections in immunocompromised patients. The alarming increase of isolates resistant to the first-line recommended antifungal therapy urges more insights into triazole-resistant *A. fumigatus* infections. In this study, we systematically optimized a longitudinal multimodal imaging-compatible neutropenic mouse model of IPA. Reproducible rates of pulmonary infection were achieved through immunosuppression (sustained neutropenia) with 150 mg/kg cyclophosphamide at day −4, −1 and 2, and an orotracheal inoculation route in both sexes. Furthermore, increased sensitivity of *in vivo* bioluminescence imaging for fungal burden detection, as early as the day after infection, was achieved by optimizing luciferin dosing and through engineering isogenic red-shifted bioluminescent *A. fumigatus* strains, one wild type and two triazole-resistant mutants. We successfully tested appropriate and inappropriate antifungal treatment scenarios *in vivo* with our optimized multimodal imaging strategy, according to the *in vitro* susceptibility of our luminescent fungal strains. Therefore, we provide novel essential mouse models with sensitive imaging tools for investigating IPA development and therapy in triazole-susceptible and triazole-resistant scenarios.

## Abstract (precis)

**Summary:** A novel reproducible longitudinal multimodal imaging-compatible neutropenic mouse model of invasive pulmonary aspergillosis provides increased early fungal detection through novel red-shifted luciferase-expressing triazole-susceptible and -resistant *Aspergillus fumigatus* strains, and boosted bioluminescence.

## INTRODUCTION

The mold *Aspergillus fumigatus* causes a spectrum of diseases in humans, ranging from allergic-bronchopulmonary infections to acute invasive pulmonary aspergillosis (IPA) (Kosmidis and Denning, 2015; Kanj et al., 2018). IPA continues to be one of the most important opportunistic life-threatening conditions in the increasing immunocompromised patient population (Kontoyiannis et al., 2010; van de Peppel et al., 2018). Despite the availability of novel or less toxic antifungals, mortality among IPA patients remains relatively high, with ∼30% of patients succumbing to infection (van de Peppel et al., 2018; Herbrecht et al., 2002). Triazoles are the recommended first-line therapy for prophylaxis and treatment of *Aspergillus*-related diseases (Tissot et al., 2017; Patterson et al., 2016). However, patient outcomes are further threatened by increasing worldwide reports of *A. fumigatus* resistance to triazole antifungals and the observed associated increased mortality among patients with resistant infections (Resendiz Sharpe et al., 2018; van der Linden et al., 2015; Resendiz-Sharpe et al., 2019; Lestrade et al., 2019). Therefore, more insights are urgently needed concerning the pathogenesis, disease development and treatment of *A. fumigatus* infections, particularly in triazole-resistant settings.

Preclinical *in vivo* studies are indispensable to characterize fungal virulence, host-pathogen interactions, dynamics of onset and progression of IPA infections and for testing novel or current antifungal therapies under controlled conditions. By far, murine animal models are the most commonly used models for studying IPA (85% of all studies), with immune responses and disease development comparable to those observed in humans (Desoubeaux and Cray, 2018). Moreover, murine models of invasive aspergillosis have provided the majority of data supporting pharmacometrics (pharmacokinetic/pharmacodynamic) and safety data, host-fungal responses and treatment recommendations in clinical settings (Desoubeaux and Cray, 2017, 2018; Lewis and Verweij, 2017; Seyedmousavi et al., 2015; Mirkov et al., 2019). However, IPA murine models vary widely and lack standardization, resulting in variable study outcomes as experimental methodologies differ in the use of strains, immunosuppressive regimens, infection routes, inoculum and disease assessment (Desoubeaux and Cray, 2017, 2018; Lewis and Verweij, 2017; Lewis and Wiederhold, 2005). In most studies, assessment of disease severity and fungal burden is performed exclusively by using invasive and variability-prone techniques, such as colony-forming unit (CFU) counting or histopathology. The statistical power of these methods, especially of CFU counts which suffer from reproducibility issues (Olsen and Bakken, 1987), is low and is typically addressed by increasing the number of animals sacrificed per timepoint, resulting in increasingly complex studies.

These issues can be overcome by implementing *in vivo* real-time imaging techniques, such as bioluminescence imaging (BLI) and micro-computed tomography (micro-CT). These techniques are non-invasive tools that provide *in vivo* longitudinal, dynamic, visual and quantitative information on fungal burden and lung lesion formation during the onset and progression of disease in individual infected animals. These implementations decrease the variability and number of animals needed per experiment (Poelmans et al., 2016, 2018; Brock et al., 2008; Galiger et al., 2013; Morisse et al., 2012). A longitudinal imaging approach is of particular interest in drug efficiency assessment studies as it allows individual and grouped therapeutic effects to be followed from the time of inoculation (Poelmans et al., 2016; Vande Velde et al., 2018). Nevertheless, *in vivo* BLI of fungal infection has been challenging, particularly in deep-seated infections (Doyle et al., 2006; Enjalbert et al., 2009). More recent reporters, such as the fungal adapted codon-optimized wild-type firefly luciferase (emission maximum at 560 nm), have been successfully used to detect deep-tissue infections in fungal infection models of mucormycosis virulence, disseminated candidiasis and cryptococcosis, and in mouse models of IPA (Poelmans et al., 2018; Galiger et al., 2013; Vanherp et al., 2019; Binder et al., 2018; Jacobsen et al., 2014). However, owing to the relatively short time course of ∼3 days from IPA infection onset to humane endpoint in neutropenic murine models (Poelmans et al., 2018; Galiger et al., 2013), detection of *A. fumigatus* burden by BLI generally occurs in the late stages of the disease development (Poelmans et al., 2018), limiting the temporal resolution of readouts and our knowledge on the infection dynamics at the early stages of disease development. To increase the sensitivity of detection of low fungal burden, particularly at earlier stages of infection, luciferases catalyzing photon emission in the red spectrum (600-700 nm), which show reduced light absorbance by hemoglobin and less tissue scattering compared to other luciferases, have been successfully implemented in *Candida albicans* and *Cryptococcus neoformans* (Vanherp et al., 2019; Dorsaz et al., 2017)*.* These luciferases have the potential to increase likewise the sensitivity of fungal burden detection in IPA, yet they have not been implemented in *A. fumigatus*. Further improvement of BLI detection sensitivity in IPA could be potentially achieved by modifying the *in vivo* luciferin doses. Studies from mammalian cells and other microorganisms reported increased bioluminescence signals in a luciferin-dose-dependent manner, which supports this idea, but to the best of our knowledge, this has not been systematically tested and applied to fungal infections (Andreu et al., 2010, 2013; Aswendt et al., 2013).

This study delivers, in a stepwise approach, a reproducible longitudinal multimodal neutropenic mouse model of IPA with increased fungal burden detection capabilities. We engineered and thoroughly characterized three bioluminescent *A. fumigatus* strains; one triazole-sensitive and two isogenic triazole-resistant strains, all expressing a red-shifted thermostable firefly luciferase. We assured the reproducible development of IPA via an optimized immunosuppressive regimen combined with an orotracheal route of inoculation that ensured sustained neutropenia for the establishment of infection and reproducible invasive pulmonary disease development. We furthermore optimized fungal detection at the early onset of disease development by the use of a red-shifted luciferase and refinement of the *in vivo* luciferin dosing. Lastly, we compared and determined the development of IPA in both male and female mice, and demonstrated the application potential of our model system in treatment experiments with liposomal amphotericin B (L-AmB) and the triazole drug posaconazole. Altogether, our study offers a powerful resource set for the investigation of IPA and drug efficacy studies in both triazole-susceptible and triazole-resistant scenarios.

## RESULTS

### Generation of triazole-susceptible and triazole-resistant *A. fumigatus* strains expressing a red-shifted firefly luciferase

To improve the sensitivity of BLI for *in vivo* detection of *A. fumigatus*, especially at the onset of disease development, we genetically engineered a triazole-sensitive and two isogenic triazole-resistant *A. fumigatus* strains (TRAF) that express a thermostable-red-shifted firefly luciferase (*luc*_OPT_red_TS_, GenScript; accession number MT554554). A red-shifted version was selected as it had already shown excellent performance when monitoring infections caused by *C. neoformans* (Vanherp et al., 2019). To induce triazole resistance, the *cyp51A* gene with a TR_34_/L98H or a TR_46_/Y121F/T289A mutation was selected, as these mutations are the two most reported mutations conferring triazole resistance in *A. fumigatus* (Resendiz Sharpe et al., 2018). At first, the luciferase-expressing triazole-sensitive *A. fumigatus* strain was generated, and served as parental strain for the triazole-resistant strains. Our novel *luc*_OPT_red_TS_ gene was cloned under the control of the constitutively active *glyceraldehyde-3-phosphate dehydrogenase* promoter (*PgpdA*) and its terminator (*TgpdA*) sequence, and was assembled with the pyrithiamine resistance gene (*ptrA*) as selection marker in fungal transformation. This entire cassette was flanked by the upstream and downstream flanking regions of the *akuB* gene to target the integration of the luciferase construct into the *akuB* locus accompanied by the deletion of the *akuB* gene. The *akuB* locus was selected to (1) express the luciferase from a defined locus and (2) to ease the subsequent generation of *cyp51A* mutant versions because the deletion of the *akuB* gene results in an increased frequency of homologous recombination without affecting pathogenicity in a murine infection model (Da Silva Ferreira et al., 2006). The *A. fumigatus* wild-type strain CBS144.89 was used for transformation to receive the Δ*akuB*::*luc*_OPT_red_TS__*ptrA* deletion cassette (Fig. S1A), and pyrithiamine was used as a selection marker. After confirmation of single-copy integration of the reporter construct into the *akuB* locus by Southern blot analysis (Fig. S2A) and initial BLI screening, the bioluminescent Δ*akuB* strain No. 5, subsequently named Af_luc_OPT_red__WT, was selected for further experiments and served as parental strain to generate the isogenic TRAF strains. To generate TRAF strains, the promoter and partial coding sequences of the wild-type *cyp51A* gene from Af_luc_OPT_red__WT was replaced by *cyp51A* gene sequences harboring either a TR_34_/L98H or TR_46_/Y121F/T289A mutation, the two most commonly reported mutations conferring triazole-resistance in *A. fumigatus* (Resendiz Sharpe et al., 2018). Transformants were selected by the addition of itraconazole to the transformation agar. Transformants were screened by Southern blot for the *in locus* integration of a single *cyp51A* replacement construct (Fig. S2B), and mutations were confirmed by gene sequencing. Transformants meeting the required criteria were named Af_luc_OPT_red__TR46 (TR_46_/Y121F/T289A) and Af_luc_OPT_red__TR34 (TR_34_/L98H), and were selected for further analysis. Lastly, to confirm that the introduced *cyp51A* gene mutations indeed conferred triazole resistance in the transformants, the wild-type *cyp51A* gene was restored in selected TRAF strains through transformation with a construct containing the entire wild-type *cyp51A* gene of the triazole-susceptible parental strain and using the hygromycin resistance cassette (Fig. S2C) as selection marker.

### *In vitro* characterization of the genetically engineered red-shifted luciferase bioluminescent *A. fumigatus* strains

To confirm that the genetic manipulations of the transformants conferred the expected triazole resistance but had no negative impact on the general performance of the strains under *in vitro* conditions, we characterized the selected strains for their triazole susceptibility, growth, sporulation, viability and bioluminescence emissions (peak total flux and wavelength emission peak). The red-shifted luciferase expressing Af_luc_OPT_red__WT, _TR34 and _TR46 strains showed comparable susceptibility phenotypes (±1 dilution difference) to the non-bioluminescent *A. fumigatus* wild-type CBS144.89 (parental strain), the bioluminescent *A. fumigatus* strain Af2/7/1 expressing a codon-optimized wild-type luciferase (Galiger et al., 2013), and the parental *cyp51A* mutation strains V-052-35 (TR_34_/L98H) and CYP-15-7 (TR_46_/Y121F/T289A), respectively (Table 1). The resistant phenotype of the transformant Af_luc_OPT__red_TR34 harboring the TR_34_/L98H mutation [itraconazole minimum inhibitory concentration (MIC) >16 mg/l] matched with the reported characteristic elevated itraconazole MIC (>4 mg/l) conferred by this mutation (Snelders et al., 2011). Similarly, the elevated voriconazole MIC of >16 mg/l of our Af_luc_OPT_red__TR46 transformant correlated with the typically reported phenotype of isolates harboring the TR_46_/Y121F/T289A mutation (voriconazole MIC >4 mg/l), with variable itraconazole and posaconazole susceptibility (Snelders et al., 2015). The complemented TRAF strains, in which the mutated *cyp51A* gene was replaced by the wild-type sequence, reverted to the triazole-sensitive phenotype (Table S3), confirming that the resistance of the engineered TRAF strains was solely due to the *cyp51A* mutations.

**Table 1.**
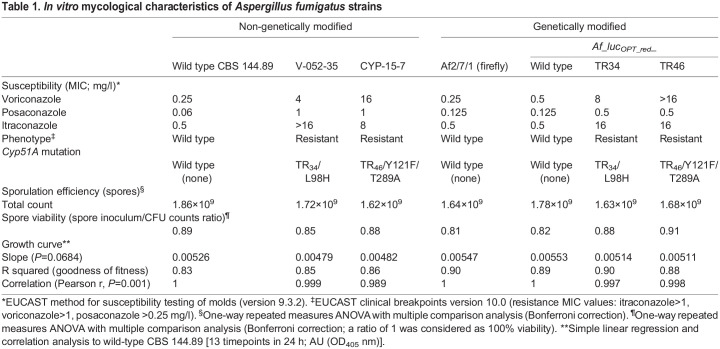
*In vitro* mycological characteristics of *Aspergillus fumigatus* strains

**Fig. 1.**
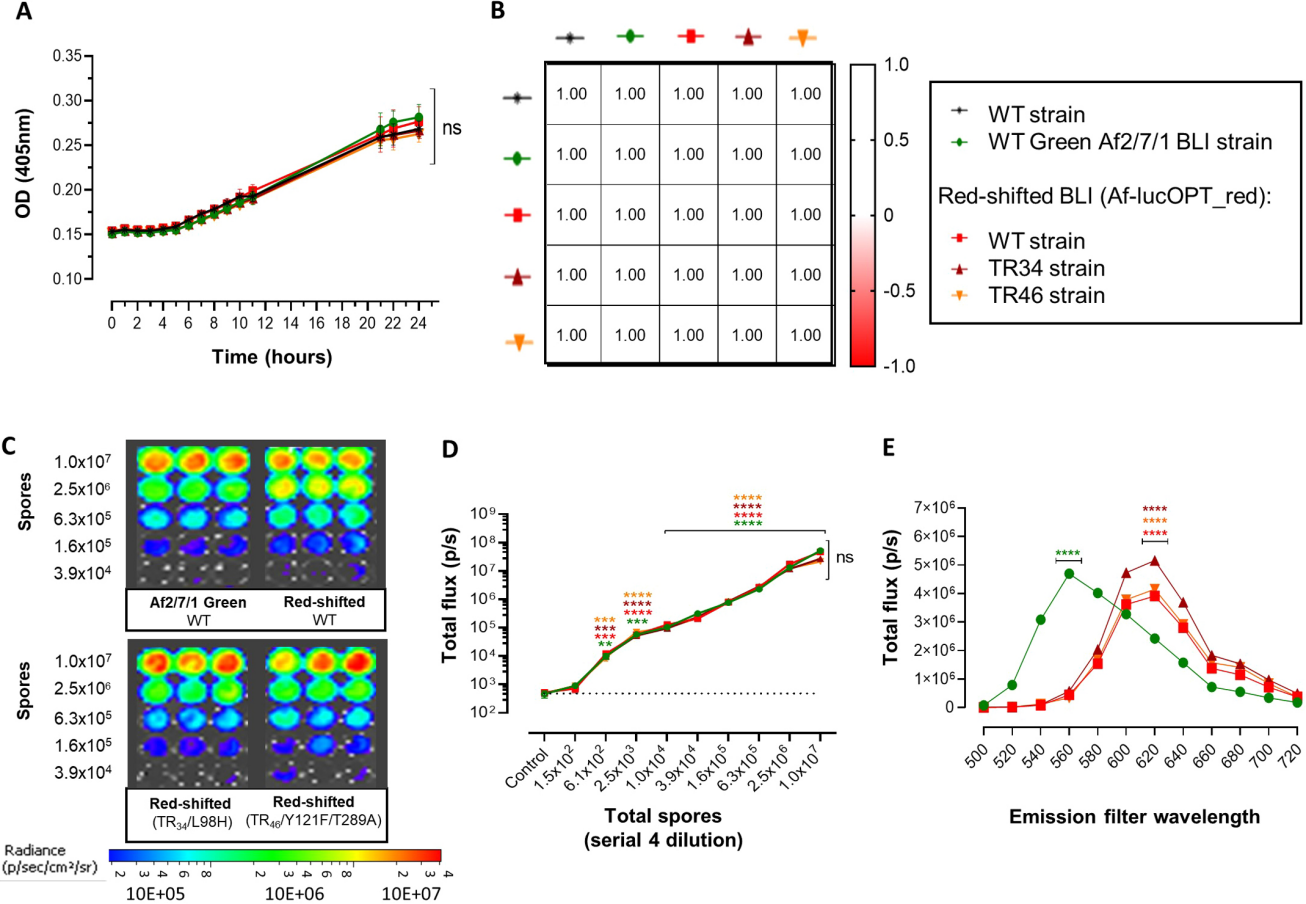
***In vitro* growth and emitted bioluminescence characterization of newly generated red-shifted luciferase-expressing *Aspergillus fumigatus* strains.** (A) Growth curve graphical representation of variations in optical density over time (24 h) of the wild-type (WT) CBS 144.89 strain (parental strain), firefly luciferase (Af2/7/1) and red-shifted firefly luciferase-expressing *A. fumigatus* strains Af_luc_OPT_red__: WT, TR34 and TR46. (B) Growth correlation heatmap matrix (Pearson r) between the wild-type parental and bioluminescent strains. (C,D) Representative image of bioluminescence (C) and peak total flux emission (D) from serial 4-fold dilutions (initial amount 1×10^7^ spores) of the Af2/7/1 and *A. fumigatus* strains expressing the red-shifted luciferase. The dotted line in the graph represents the control measurement (no spores; luciferin plus PBS). Quantification of bioluminescence showed that cells could be detected starting from 610 spores per well (LOD). (E) Total flux spectral emission of bioluminescent strains (emission filters 500 to 720 nm wavelength; inoculum 1×10^7^ spores) and peak total flux emission comparison between the Af2/7/1 (green spectrum) and the red-shifted luciferase *A. fumigatus* strains. Data are mean±s.d. (*n*=5). **P≤0.001; ***P≤0.0001; ****P≤0.0001 [simple linear regression analysis and one-way repeated measures ANOVA (multiple comparison analysis; Tukey's method)]. ns, not significant.

There were no differences in strain sporulation efficiencies [1.71×10^9^±8.54×10^7^ (mean±s.d.) total spores per cultured flask; *P=*0.1271] after 4-day incubation or in spore viability from initial inoculum [0.86±0.037 (mean±s.d.) spore inoculum/CFU counts ratio, *P=*0.1386] between our transformants and the wild-type CBS 144.89 reference strain (Table 1). Similarly, 24-h growth rate comparison analysis showed no significant differences in growth (simple linear regression analysis; slope *P*=0.0684, x-y intercept *P*=0.2294) and a large strength of association of growth between strains [Pearson correlation r=0.998 (range 0.989-1), Table 1, Fig. 1A,B).

Bioluminescence signals of all of the red-shifted *A. fumigatus* strains showed similar peak light emissions that significantly increased with the inoculum concentration (Fig. 1C,D), and were, likewise, comparable to bioluminescence signals from the previously characterized Af2/7/1 bioluminescent strain (*P=*0.2997; Galiger et al., 2013). Quantification of bioluminescence signals from a 4-fold serial dilution of non-germinated conidia (Fig. 1D) detected fungal spores from an inoculum range of 1×10^7^ to 6.1×10^2^ spores [total flux (p/s): 4.7×10^3^±1.1×10^3^ (mean±s.d.); *P*=0.0147]. This brings the limit of detection (LOD) down to 610 in all bioluminescence strains that can be detected without prior activation in growth medium above background when compared to control measurements (luciferin plus PBS without spores). Spectral imaging confirmed the red-shifted spectrum of the emitted light from the newly generated strains with maximum peak total flux emissions in the 620-nm red-spectrum emission filter [total flux (p/s): wild type, 3.91×10^6^; TR_34_/L98H, 5.16×10^6^; TR_46_/Y121F/T289A, 4.16×10^6^) compared to the observed peak total fluxes of the Af2/7/1 strain in the 560-nm green spectrum emission filter (total flux: wild type, 4.69×10^6^) (Fig. 1E). These analyses confirmed that the expression of the luciferase in the *akuB* locus and the modification of the *cyp51A* gene cause no obvious phenotypes under *in vitro* conditions except the expected triazole resistance in the strains harboring the modified *cyp51A* genes.

### Optimized immunosuppression induction in mice by varying the cyclophosphamide regimen

**Fig. 2.**
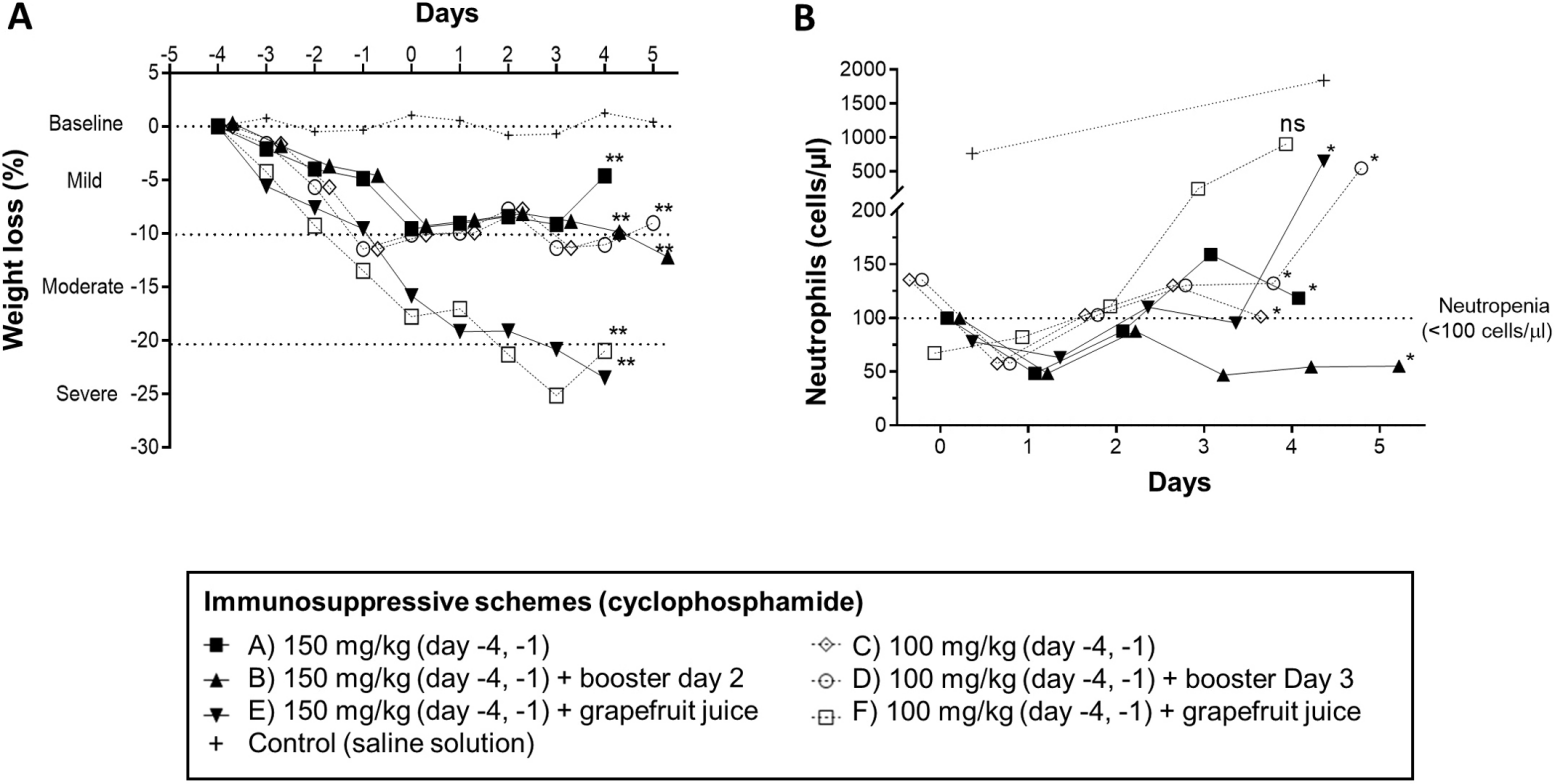
**Comparison of different cyclophosphamide immunosuppressive scheme effects on weight loss and neutropenia induction in mice.** (A) Evolution of weight loss (percentage compared to baseline; mild, 0-10%; moderate, 10-20%; severe, >20%) under different tested cyclophosphamide immunosuppressive schemes and control group (saline solution) from day −4 (initiation of immunosuppressive therapy/baseline) until day 4 or day 5 in booster scheme groups. Graph represents the mean value of weight loss. (B) Neutrophil count (cells/µl) kinetics after cyclophosphamide-induced immunosuppression. Graph represents the mean value (*n*=3 males per group per timepoint). *P≤0.05; **P≤0.005; ns, non-significant [mixed model ANOVA (multiple comparison analysis, Tukey's correction) to control group].

The establishment of IPA infections generally depends on the immune status of the host and relates to the duration and degree of immunosuppression (neutropenia) (Kalleda et al., 2016). However, only a few studies differing in immunosuppressive drugs used, animal gender, dosing and timing have monitored their effects on neutrophil population kinetics (Brunck et al., 2014; Zuluaga et al., 2006). Here, we evaluated the daily effects of two commonly used doses of cyclophosphamide (100 or 150 mg/kg dose; leucopenia model) on weight loss, survival and blood cell counts in pre-established conditions (regimens A-F; Fig. 2), to determine the most suitable regimen that warrants sustained neutropenia and the establishment of infection (IPA) without severely compromising mouse health (i.e. severe weight loss of >20%). Neutrophil recovery was observed in all 100 mg/kg regimes (as early as day 2) and in regimen A on day 3, with mean±s.d. neutrophil counts of 142.5±157 cells/μl and 159±52 cells/μl, respectively (Fig. 2A). The immunosuppressive regimen of cyclophosphamide at a dose of 150 mg/kg [days −4 and −1 with a booster at day 2 (regimen B)] resulted in continuous neutropenia (<100 cells/µl) and weight loss within the accepted range (<20% weight loss), until the end of the experiment on day 5 (Fig. 2A,B) in male mice. An overview of mean cell counts per group and immunosuppressive regimen is depicted in Table S1. Of note, cyclophosphamide is metabolized by hepatic cytochrome P450 enzymes, and its concomitant administration with other substances may affect its metabolization, increasing adverse reactions or altering its immunosuppressive effects. Such alterations could be observed with the simultaneous administration of grapefruit juice. Grapefruit juice, a known inhibitor of cytochrome P450 enzymes, is given to reduce the rapid metabolization of certain drugs in mice, such as the triazole antifungal voriconazole (Sugar and Liu, 2000). Considering that voriconazole can be potentially used in IPA cyclophosphamide-induced neutropenic murine models, we also determined the concomitant effects of grapefruit juice and cyclophosphamide (regimens E and F; Fig. 2A,B) on weight and neutropenia induction. In this setting, the majority of mice from both cyclophosphamide dose regimes with concomitant grapefruit juice administration (72%, 13/18) reached severe weight loss (>20%, humane endpoint) by day 3 and needed to be sacrificed (28% survival), strongly contraindicating the co-administration of grapefruit juice in these immunosuppressive regimens.

Next, to exclude potential gender-related effects on immunosuppression development, we determined whether the observed immunosuppressive effects of the cyclophosphamide regimen of 150 mg/kg (day −4 and −1) with a booster at day 2 could be reproduced in female mice. Comparably, this regimen achieved persistent neutropenia and weight loss within the accepted range until the end of the experiment on day 5 in female mice (Fig. S3), corroborating the immunosuppressive effects of this regimen in mice of either sex, allowing the establishment of infection (IPA) without severely compromising mouse health. For these reasons, this regimen was chosen for all subsequent experiments.

### IPA development assessment upon infection with the novel red-shifted luciferase expressing wild-type *A. fumigatus* strain

**Fig. 3.**
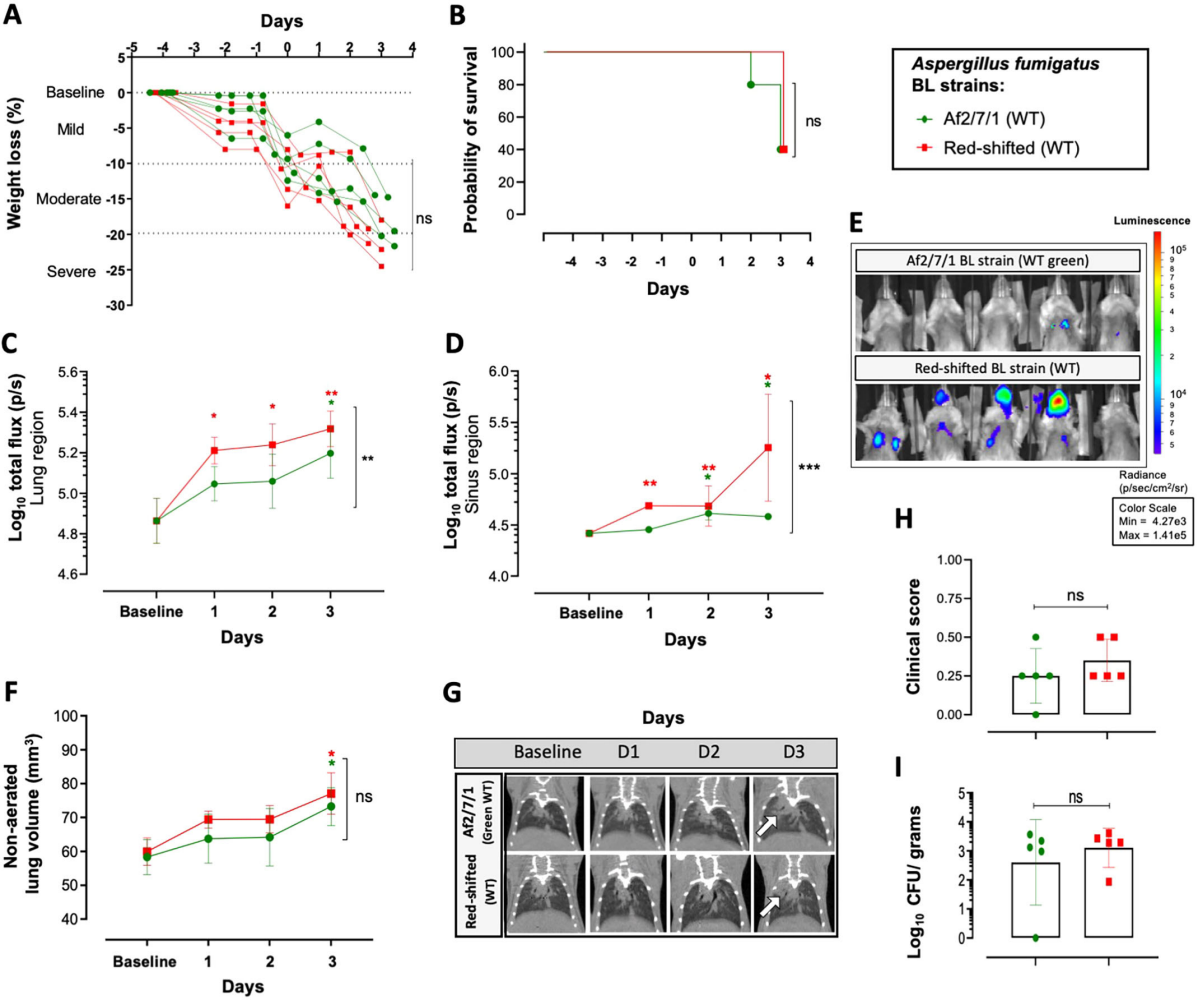
**Multimodal *in vivo* IPA development assessment of the newly generated wild-type *Aspergillus fumigatus* strain expressing red-shifted luciferase.** (A,B) Comparison of weight evolution (percentage weight loss compared to baseline) (A) and of survival of mice infected with the wild-type (WT) red-shifted luciferase Af_luc_OPT_red__WT *A. fumigatus* strain and the previously characterized codon-optimized firefly bioluminescent (BL) Af2/7/1 *A. fumigatus* strain upon intranasal infection (5×10^5^ spores; *n*=5 per group) (B). (C,D) Graph representing the total flux (log_10_) quantified over time from bioluminescence images of the lung (C) and sinus regions (D). (E) Representative bioluminescent images depicting mice fungal bioluminescence burden progression on day 3 after inoculation. (F) Graphs representing the non-aerated lung volume (lung lesions) of infected groups. (G,H) Longitudinal micro-CT illustrative images of lung lesion development from each infected group (white arrow depicts pulmonary infiltrates) (G) and mean cumulative image clinical score from micro-CT-derived data based on lung lesion progression over time (H). (I) CFU counts (log_10_ CFU/g counts) from lung homogenates from each mouse per group at sacrifice, day 3 after inoculation. Data are mean±s.d. of the results from multiple mice (*n*=5 male). *P≤0.05, **P≤0.005, ***P≤0.0005 to baseline (above) and between groups (lateral) [two-way repeated measures ANOVA (multiple comparison analysis, Tukey's correction) (A,C,D,F), log-rank (Mantel–Cox) test (B) and two-tailed paired Student's *t*-test (H,I)]. ns, not significant. Boxes in graphs represent the mean from each group.

We subsequently determined the ability of the Af_luc_OPT_red__WT strain to develop IPA *in vivo* and analyzed the sensitivity of detection of the fungal burden by BLI in neutropenic infected mice (intranasal inoculation, 5×10^5^ spores). These analyses were compared to those from the previously validated *A. fumigatus* wild-type strain Af2/1/7 expressing a randomly integrated luciferase emitting light in the green-spectrum (Poelmans et al., 2018; Galiger et al., 2013). A longitudinal multimodal approach consisting of daily weight and survival assessment, BLI signal amplitude (total flux) and lung lesion (micro-CT) determination, in addition to CFU counts at the end of the experiment, was used. Weight loss (*P*=0.2795) and survival (*P*=0.7655) (Fig. 3A,B) were similar for both groups. Compared to the baseline, the BLI signal from the lung was significantly increased (*P*=0.0474) in the red-shifted wild-type group starting at day 1 (Fig. 3C,E). Further, compared to the Af2/7/1 group, the BLI signals from the lung of the red-shifted wild-type group were likewise significantly higher on day 1 (*P*=0.0013). Importantly, this intranasal infection model resulted in BLI signals from the sinus region that were significantly higher in Af_luc_OPT_red__WT infected mice on day 3 compared to the Af2/7/1 group, and were detectable from day 1 when compared to the baseline (Fig. 3D). This indicates that a significant number of spores from the inoculation can get trapped in the sinus region, which makes reproducible lung infection by the intranasal route challenging. Nevertheless, development of lung lesions over time, established by quantitative analysis of the micro-CT-derived non-aerated lung volume, was similar between both infected mouse groups (mm^3^, *P*=0.4310; percentage, *P*=0.5944) (Fig. 3F; Fig. S4E) and became significantly elevated compared to baseline measurements on day 3. Quantification of the visual assessment of micro-CT scans revealed likewise comparable lung lesion development between both groups, as further supported by similar clinical scores for both groups (0.25±0.15 points, *P*=0.3466; Fig. 3G,H). No significant differences were observed in the overall density of the lung and in aerated or total lung volume (lesions and air volume modifications) between infected mice from both groups, further suggesting a comparable lung disease progression with both *A. fumigatus* strains (Fig. S4A-D). CFU counts from lung homogenates to determine fungal burden were likewise comparable between groups (Fig. 3I; *P*=0.6768). We can conclude that our newly developed Af_luc_OPT_red__WT strain has superior and earlier *in vivo* bioluminescent signal detection capabilities compared to the Af2/7/1 green strain, without apparent differences in disease development.

### Orotracheal route of inoculation for increased reproducibility of IPA development in neutropenic mice

Our *in vivo* BLI analyses from intranasal infection revealed significant sinus infections in some animals, confirming observations from previous studies (Poelmans et al., 2018; Galiger et al., 2013). The development of prominent sinus infections in some intranasally infected mice raises concerns about the reproducibility of pulmonary aspergillosis in this infection model. Therefore, we compared an orotracheal instillation route with the intranasal route of infection with our longitudinal multimodal imaging approach to determine the most suitable route for consistent pulmonary infection.

In a first step, we optimized anesthesia induction in immunosuppressed sham-infected mice. Neutropenic orotracheally sham-infected mice lost significantly more weight when anesthesia was induced by i.p. injection (>15%, 3/5 mice; *P*=0.0269) compared to gas anesthesia-induced mice (Fig. S5A). Therefore, all subsequent orotracheal inoculations were performed via inhaled anesthesia. We next confirmed a successful bilateral orotracheal inoculation technique (*n*=4 healthy mice) by visually detecting orotracheally instilled trypan blue (20 µl) in both lungs (Fig. S5B).

**Fig. 4.**
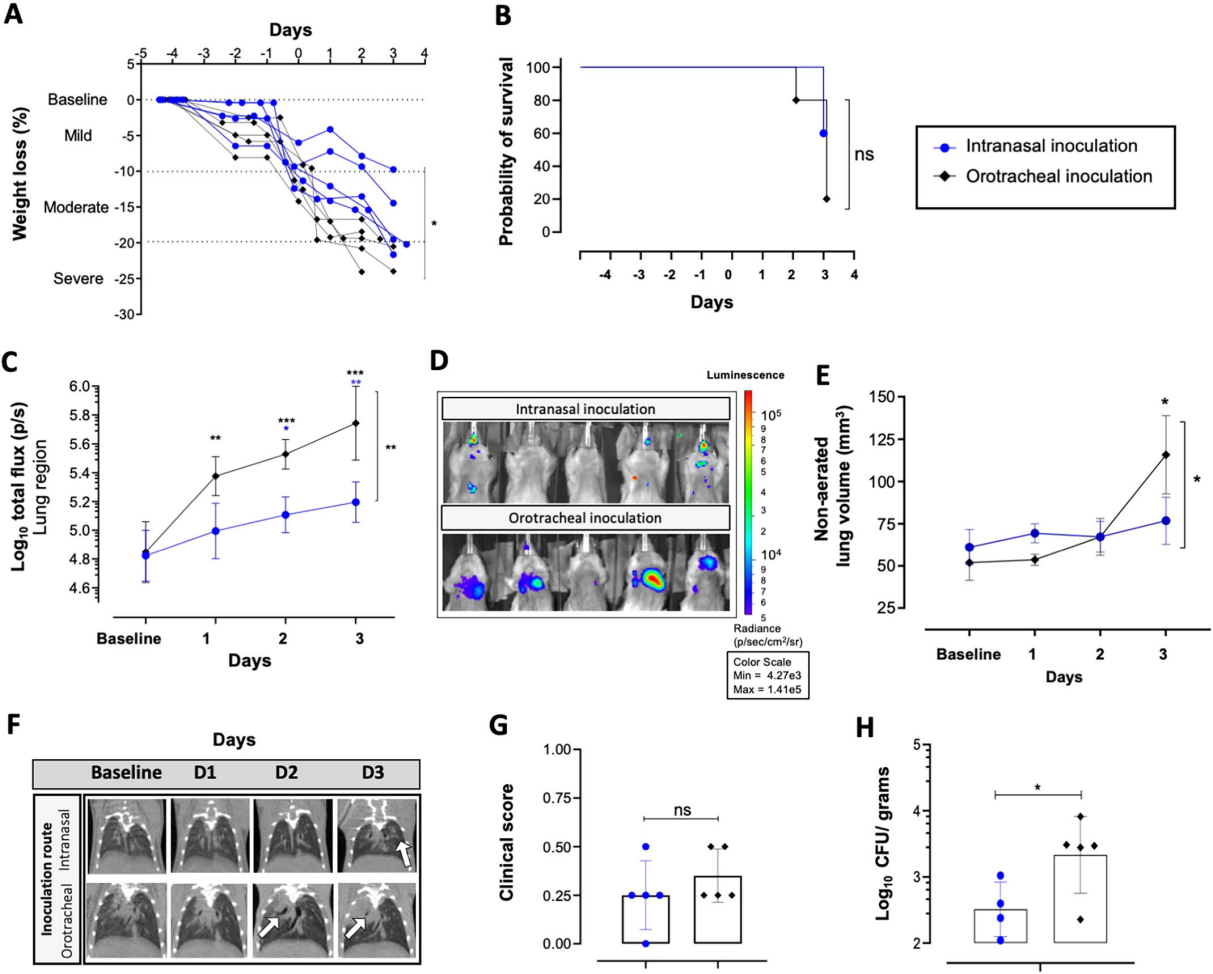
**Development of IPA following intranasal versus orotracheal inoculation with the wild-type red-shifted luciferase-expressing *Aspergillus fumigatus* strain.** (A,B) Graphical representation of weight loss percentage evolution comparison (A) and survival (B) after intranasal and orotracheal inoculation (inhaled anesthesia) with the red-shifted luciferase wild-type *A. fumigatus* strain (5×10^5^ spores; *n*=5 per group). (C,D) Longitudinal quantification of bioluminescent signal (log_10_ total flux) of pulmonary ROI in intranasally and orotracheally inoculated mice, and representative bioluminescent images on day 3 after infection (D). (E) Non-aerated lung volume (lung lesions development) quantified from lung micro-CT scans of infected mice before (baseline) and after inoculation. (F,G) Representative longitudinal scan images from the lung region (white arrows denote site of lung lesions) (F) and cumulative visual clinical score of lung lesion development based on micro-CT lung scans (G). (H) Fungal burden assessed by CFU counts (log_10_ CFU/g) from lung homogenates at day 3 after intranasal or orotracheal inoculation. Data are mean±s.d. *n*=5 males. *P≤0.05, **P≤0.005, ***P≤0.0005 to baseline (above) and between groups (lateral) [two-way repeated measures ANOVA (multiple comparison analysis, Tukey's correction; A,C,G), log-rank (Mantel–Cox) test (B) and two-tailed paired Student's *t*-test (E,H)]. ns, not significant. Boxes in graphs represent the mean from each group.

Subsequently, immunosuppressed mice were orotracheally inoculated with the Af_luc_OPT_red__WT strain (5×10^5^ spores). When compared to intranasally inoculated mice (Af_luc_OPT_red__WT strain 5×10^5^ spores), pulmonary fungal burden represented by BLI signals (total flux) was significantly higher in the orotracheally instilled group (*P*=0.0015) from day 1 post-infection (*P*=0.0037) compared to the intranasal route group, which had a BLI signal detectable above baseline only at day 2 post-infection (Fig. 4C,D). Lung lesion development quantified by micro-CT revealed significantly increased non-aerated lung volume (mm^3^ *P*=0.0106; % *P*=0.002) in the orotracheally inoculated group compared to intranasally infected mice (Fig. 4E; Fig. S5G). Other micro-CT-derived biomarkers corroborated the increased presence of lung lesions, with increased total volume (*P*=0.8782) and mean density (*P*=0.0035), and decreased lung aerated volume percentage (*P*=0.0001) on day 3 compared to baseline scans (Fig. S5C-G). A trend of increased visual lesion development (micro-CT visual clinical score; Fig. 4F,G) was also observed in the orotracheally infected group (mean 0.35±0.15; mean±s.d.) compared to intranasal infection (0.25±0.18; mean±s.d.; *P*=0.3214). Lung CFU counts at endpoint were significantly increased in the orotracheally instilled group (*P*=0.0484; Fig. 4H). Based on these results, an orotracheal inoculation under inhaled anesthesia limited the co-morbidity from anesthesia induction in mice after inoculation, and favored the development of a pulmonary infection, as inferred by decreased survival (*P*=0.1172; Fig. 4B), increased weight loss (percentage; Fig. 4A) and lung lesions in micro-CT biomarkers, along with higher pulmonary fungal burdens in CFU counts and BL signals.

To further validate the use of our imaging biomarkers of bioluminescence signal (total flux and fungal burden) and micro-CT lung lesion development [non-aerated volume (mm^3^) and cumulative clinical score] in our longitudinal non-invasive tracking of IPA development, we correlated the acquired biomarker data from this study section with CFU counts, which are currently used as the gold standard for determining fungal burden and disease development. Bioluminescence imaging (total flux and fungal burden) showed a strong correlation (Akoglu, 2018) (r=0.7168; *P*=0.0492) with lung CFU/g (Fig. S5H). Likewise, a good correlation between CFU counts/g and micro-CT non-aerated volume (r=0.6414, *P*=0.0313; Fig. S5I), and cumulative clinical score (r=0.6654, *P*=0.0357; Fig. S5J), was observed. Thus, the correlation between our imaging biomarker readouts and CFU counts provides further support for the use of BLI and micro-CT for the longitudinal monitoring of IPA development.

Overall, we can conclude that an orotracheal inoculation under inhaled anesthesia limited the co-morbidity from anesthesia induction in mice after inoculation, and favored the development of a pulmonary infection, as inferred from increased lung lesions in micro-CT biomarkers and higher pulmonary fungal burdens in CFU counts and bioluminescence signals.

### Multimodal *in vivo* imaging of IPA development from triazole-resistant *A. fumigatus* strains expressing the red-shifted luciferase

**Fig. 5.**
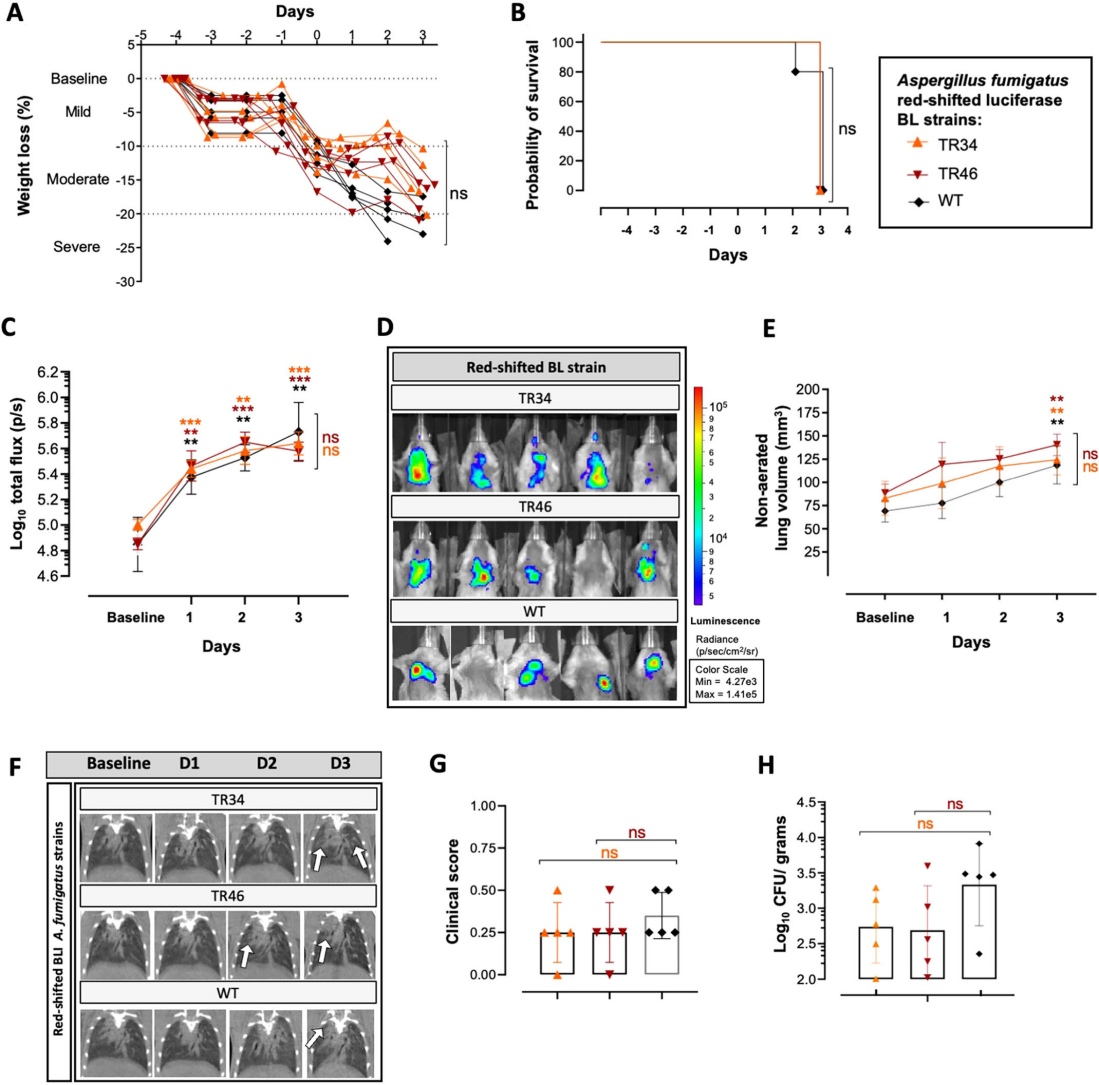
***In vivo* multimodal disease development analysis of triazole-resistant *Aspergillus fumigatus* strains expressing the luciferase in the red spectrum.** (A,B) Weight loss percentage (A) and survival comparison (B) of orotracheally inoculated mice (5×10^5^ spores) with red-shifted luciferase-expressing strains harboring the TR_34_/L98H (Af_luc_OPT_red__TR34) and TR_46_/Y121F/T289A (Af_luc_OPT_red__TR46) triazole-resistant mutations and the wild-type (WT) (Af_luc_OPT_red__WT) strain before (baseline) and after infection (*n*=5 per group). (C,D) Longitudinal bioluminescence signals (log_10_ total flux) from pulmonary ROI of infected mice (C) and representative bioluminescence images on day 3 after inoculation (D). (E) Quantitative evaluation of lung lesion development (non-aerated lung volume) after infection. (F,G) Representative coronal pulmonary micro-CT images of infected groups (arrows denote site of lesions) (F) and cumulative clinical scores representing visual assessment of lung lesion development on micro-CT scans (G). (H) CFU counts (log_10_ CFU/g) from lung homogenates of infected mice at sacrifice (day 3 after inoculation). Data are mean±s.d. *n*=5 male. **P≤0.005, ***P≤0.0005 to baseline (above) and to wild-type group (lateral) [two-way repeated measures ANOVA (multiple comparison analysis, Tukey's correction; A,C,E,G,H), log-rank (Mantel–Cox) test (B)]. ns, not significant. BL, bioluminescent.

To confirm the ability of the newly generated Af_luc_OPT_red__TR34 and Af_luc_OPT_red__TR46 TRAFs to cause IPA, we analyzed their longitudinal detectability by multimodal imaging with BLI and micro-CT after orotracheal inoculation (Fig. 5). As with fungal burden detection from mice infected with the red-shifted wild-type bioluminescence strain, both TRAF-infected mouse groups [Af_luc_OPT_red__TR34 (*P*≤0.0001) and Af_luc_OPT_red__TR46 (*P*=0.0004)] showed significantly increased bioluminescence emission compared to baseline scans (Fig. 5C,D) from day 1 post-infection, without significant difference among groups and with no significant differences in weight loss or survival (Fig. 5A,B). Comparable to the wild-type strain, lung lesion development (non-aerated lung volume; mm^3^ and percentage) in both TRAF stains was likewise significantly detectable on day 3 (Fig. 5E; Fig. S6E), with increased total volume mean density and decreased aerated (%) lung volume (*P*=0.0500; Fig. S6C,D). In addition, neither red-shifted TRAF-infected mice showed significant differences in other micro-CT-derived lung biomarkers (Fig. S6A,B), clinical visual lesion development (Fig. 5F,G) and CFU counts (Fig. 5H), reflecting a similar disease progression. In conclusion, compared to the wild-type red-shifted luciferase expressing strain, the novel TRAF strains presented similar early *in vivo* fungal burden detection capabilities (BLI) with comparable disease development.

### Effect of increased luciferin dosing on the sensitivity of *in vivo* BLI at early and later stages of infection

**Fig. 6.**
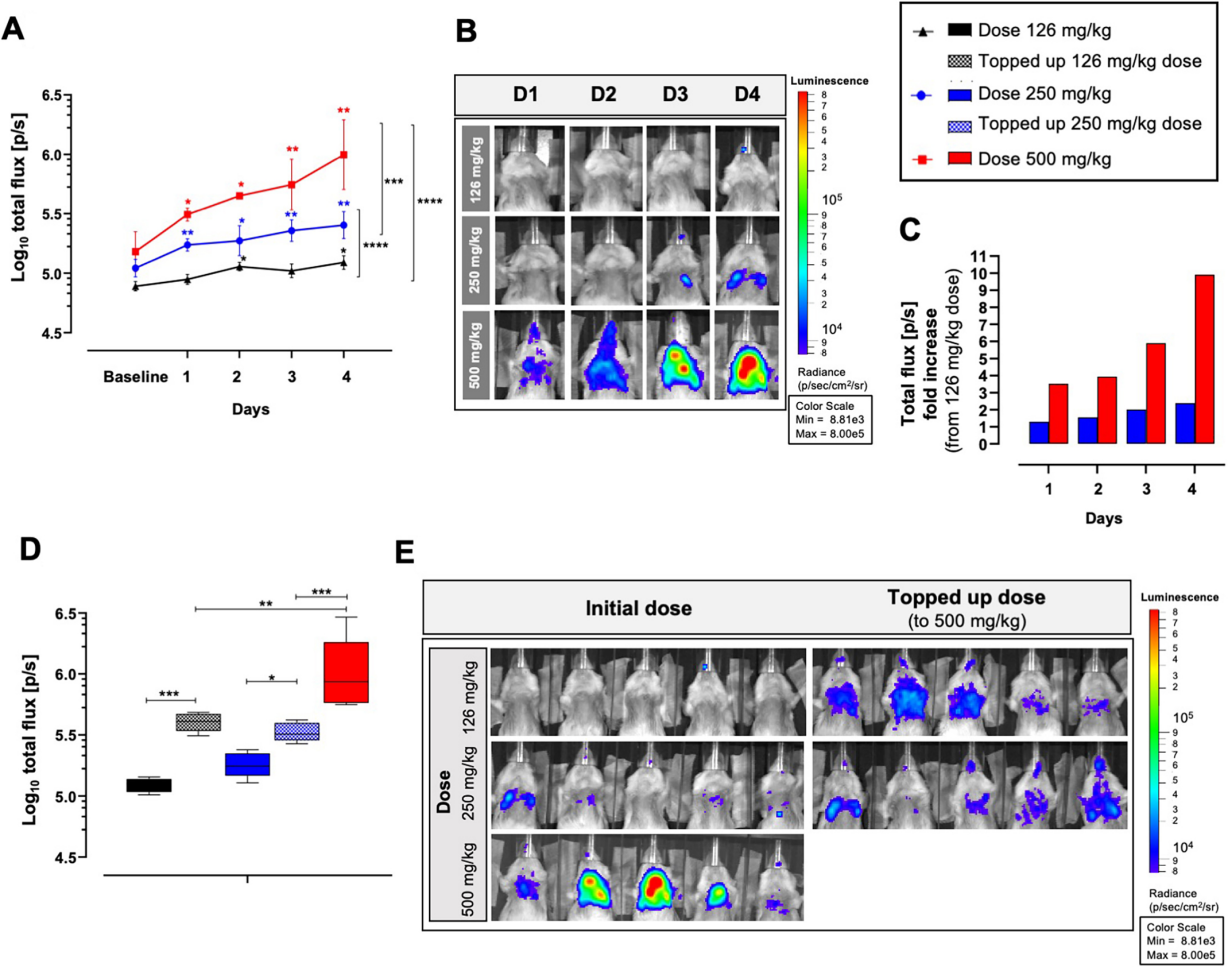
**Luciferin dose-dependent total flux comparison of *in vivo* bioluminescence imaging of *Aspergillus fumigatus* infected mice.** (A,B) Graph representing the total photon flux (log_10_) quantified from bioluminescence images from an ROI (lung region) of orotracheally inoculated mice (5×10^5^ Af_luc_OPT_red__WT spores) according to administered luciferin dose (*n*=5 per group) (A) and corresponding representative longitudinal bioluminescence images after inoculation (B). (C) Fold increase of total flux (p/s) of infected mice after administration of the 250-mg/kg and the 500-mg/kg luciferin doses compared to the 126-mg/kg dose (from mean group values). (D) Graph representing total log_10_ photon flux comparison of infected mice from the lung region after complementation of initial luciferin dose to 500 mg/kg quantified on day 4 after infection (solid bars represent mice total fluxes from initial luciferin doses; non-solid bars represent total fluxes after complementation with a 500 mg/kg luciferin dose). (E) Representative bioluminescence images of infected mice (including one mouse from each condition also shown in panel B, day 4) after administration of initial (all doses) and supplemented (126 mg/kg and 250 mg/kg) luciferin doses. Data are mean±s.d. *n*=5 males*.* *P≤0.05, **P≤0.005, ***P≤0.0005, ****P≤0.0001 to baseline (above) and between groups (lateral) [two-way repeated measures ANOVA (multiple comparison analysis, Tukey's correction)].

To determine whether the fungal burden detection sensitivity can be increased as with other non-fungal microorganisms (Andreu et al., 2010, 2013; Aswendt et al., 2013), we next explored the effect of higher dosages of luciferin in the *in vivo* luciferin application on the sensitivity of detection of pulmonary aspergillosis. An increase was of particular interest for the early stages of disease development (i.e. the very first 48 h post-infection) in our murine IPA model, during which the bioluminescence signals were only slightly above background levels. Neutropenic mice were orotracheally inoculated with 5×10^5^ Af_luc_OPT_red__WT spores and followed up for disease development by longitudinal multimodal imaging. Fungal burden detection by BLI was longitudinally assessed in mice using three different luciferin doses (i.p. injection): 126 mg/kg (standard reported dose) (Poelmans et al., 2018; Vanherp et al., 2019; Lee et al., 2003), 250 mg/kg and 500 mg/kg. The increase to a 250-mg/kg dose produced a 1.3 to 2.2-fold total flux (p/s) increase from day 1 to day 4 post-infection compared to the 126-mg/kg dose (*P*=0.0001; Fig. 6A-C). The 500-mg/kg dose increased total photon fluxes by 2-fold compared to the 126-mg/kg dose on day 1 post-infection and by ∼1 log (9.3-fold increase) on day 4 (*P*≤0.0001). Importantly, fungal infections were significantly detectable as early as day 1 after infection at doses of 250 mg/kg (*P*=0.0071) and 500 mg/kg (*P*=0.0455) compared to day 2 with the 126 mg/kg dose (*P*=0.0166). Bioluminescence signals from the 500-mg/kg regimen were significantly higher than those from the 250-mg/kg dose (*P*=0.0003).

Boosting the sensitivity of BLI for detecting aspergillosis was further confirmed by topping-up doses to a total concentration of 500 mg/kg in both the 126 mg/kg and 250 mg/kg luciferin dose-groups on day 4 (Fig. 6D,E). In the group administered with an initial 126-mg/kg luciferin dose, topping-up the initial dose to a total dose concentration of 500 mg/kg resulted in a significant increase in total photon flux of ∼2.2-fold (*P*=0.0003). Likewise, complementing the initial dose of the 250-mg/kg group to a 500-mg/kg dose resulted in a significantly increased BLI signal (1.8-fold increase) compared to the initial dose (*P*=0.008), but never reached the signal intensities from the direct application of 500 mg/kg, which may be due to rapid uptake and elimination of luciferin from the first injection by the intestine after i.p. injection (Berger et al., 2008). We verified that differences in total fluxes between groups could not be explained by variations in fungal burdens between groups, as comparable disease development was observed in lung micro-CT biomarkers and from CFU counts (Fig. S7C-H). Furthermore, there appeared to be no associated luciferin dose toxicity, as weight loss and survival were comparable among all luciferin-dose groups (Fig. S7A,B). This study indicates that a dose of 500 mg/kg luciferin results in significantly increased total photon fluxes in lung-infected mice compared to other doses, with no indications of toxicity, enabling sensitive detection of lung infection as early as the first day after inoculation.

### Comparison of *in vivo* IPA development between male and female mice by multimodal imaging

We next applied our model to determine the possibility of sex-based differences in the development of IPA in cyclophosphamide-immunosuppressed infected mice. For this, male and female mice (*n*=3 per group) were followed longitudinally using our multimodal imaging approach after orotracheal inoculation with the red-shifted luciferase-expressing strains: Af_luc_OPT_red__WT, _TR34 and _TR46 (5×10^5^ spores; luciferin dose of 500 mg/kg). No significant sex-related differences in weight loss and survival were observed in any of the inoculated groups (Fig. S8A,B). Likewise, no significant differences in fungal burden were detected in infected mice, as similar bioluminescence emissions between male and female mice were observed over the time course of the experiment (Fig. S8C,D). Furthermore, all inoculated groups presented significantly increased bioluminescence signals compared to baseline scans from day 1 post-infection regardless of sex [males, *P*=0.0242 (wild type), 0.0200 (TR_34_), 0.0500 (TR_46_); females, *P*=0.0120 (wild type), 0.0461 (TR_34_), 0.0157 (TR_46_) on day 1]. Similar to infected male mice, lung lesion development (non-aerated lung volume; mm^3^) in female mice increased over time compared to baseline scans [males *P*=0.0022 (WT), 0.0022 (TR_34_), 0.0005 (TR_46_); females *P*=0.0090 (WT), 0.0489 (TR_34_), 0.0375 (TR_46_) on day 3], with no significant differences in µCT-derived scores (Fig. S8E-G) or CFU counts (Fig. S8H) between infected mice. In summary, the development of IPA does not significantly differ in our model between female and male mice.

### Multimodal assessment of antifungal treatment efficacy on IPA development *in vivo*

Finally, we assessed the potential and added value for *in vivo* use of our new luciferase-expressing *A. fumigatus* strains, and the accompanied multimodal imaging approaches, in drug treatment studies for IPA. For this purpose, we compared the antifungal efficacies of posaconazole, L-AmB and placebo treatment on the development of IPA after infection with our red luciferase-expressing triazole-susceptible (Af_luc_OPT_red__WT) and TRAF strains (Af_luc_OPT_red__TR34 and Af_luc_OPT_red__TR46).

**Fig. 7.**
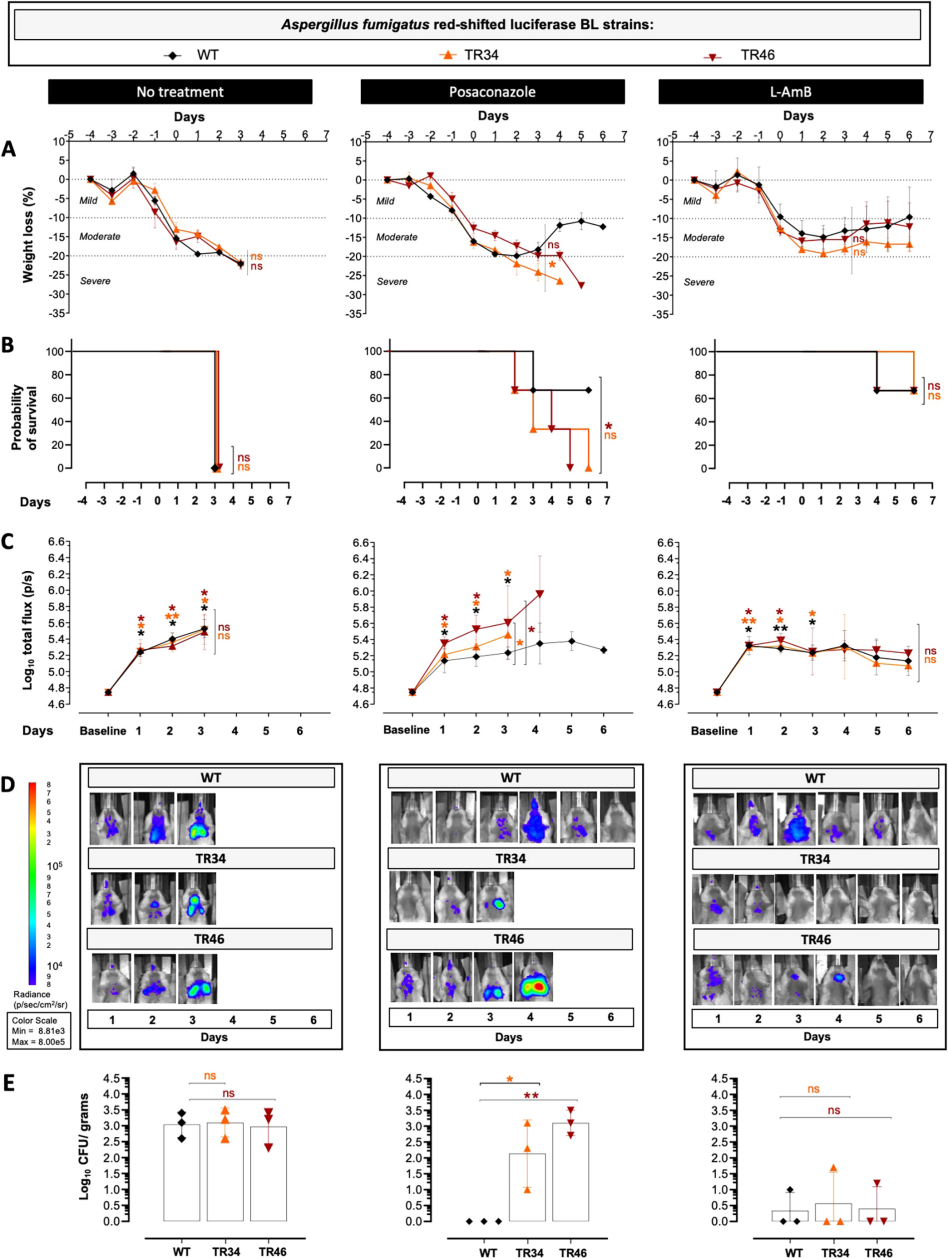
***In vivo* antifungal treatment efficacy assessment of triazole-susceptible and -resistant *A. fumigatus* infected mice.** *In vivo* monitoring of placebo (saline solution, left), posaconazole (middle) and L-AmB (right) therapy efficacies on IPA development in cyclophosphamide-immunosuppressed mice after infection with 5×10^5^ spores from the triazole-susceptible (Af_luc_OPT_red__WT) and the triazole-resistant (Af_luc_OPT_red__TR34, Af_luc_OPT_red__TR46) red-shifted luciferase-expressing *A. fumigatus* strains. (A,B) Longitudinal weight loss percentage (A) and survival comparison of treated mice (B). (C,D) Bioluminescence signal quantification (log_10_ total flux) from the lung region of infected mice before (baseline) and after infection (C), and corresponding representative bioluminescence images (D). (E) CFU counts from lung homogenates (log_10_ CFU/g) after treatment of infected mice at day 6 or at humane endpoint. Data are mean±s.d. *n*=3 males. ns=not significant, *P≤0.05, **P≤0.005 to baseline (above) and to no treatment group (lateral) [two-way repeated measures ANOVA (multiple comparison analysis, Tukey's correction; A,C,E), Log-rank (Mantel–Cox) test (B)]. ns, not significant. BL, bioluminescent; WT, wild type.

The triazole posaconazole is recommended for prophylaxis and therapy of IPA (Tissot et al., 2017; Patterson et al., 2016), and was selected because it has high oral bioavailability in mice (Seyedmousavi et al., 2015) and can be potentially used without concomitant use of grapefruit juice (recommended for voriconazole treatment) (Sugar and Liu, 2000). Starting from 1 h after orotracheal infection, cyclophosphamide-immunosuppressed mice were treated daily with either posaconazole (8 mg/kg dose, oral gavage), L-AmB [10 mg/kg dose; intraperitoneally (i.p.)] or placebo (saline solution; i.p.). Disease development was followed longitudinally in each mouse with our multimodal imaging approach for up to 6 days or until humane endpoints were reached. As expected, regardless of the inoculated strain, all mice treated with placebo presented with moderate to severe weight loss (Fig. 7A, left) and a decrease in survival (3/3; Fig. 7B, left) within a 3-day timeframe post-infection, with no significant differences in terms of the *A. fumigatus* strain used for infection. IPA development in all placebo-treated mice was characterized by comparable significantly increasing bioluminescence signals from day 1 post-infection compared to baseline scans [*P*=0.0023 (wild type), 0.0314 (TR_34_), 0.0500 (TR_46)_] and equivalent CFU counts on day 3 (Fig. 7C-E, left). Lung lesion development, as determined by micro-CT biomarkers, was likewise significantly increased and comparable between all infected mice (Fig. S9), demonstrating altogether that placebo treatment (saline solution) does not modify the previously observed development of IPA after infection with either strain.

With respect to posaconazole treatment, mice infected with the triazole-susceptible bioluminescent strain (wild type) revealed an initial weight loss followed by subsequent weight gain starting from day 3 after infection (Fig. 7A, middle). However, this was not the case for mice infected with either of our triazole-resistant strains (Af_luc_OPT_red__TR34 and Af_luc_OPT_red__TR46), in which weight loss continued over time compared to wild-type-infected mice [*P*=0.0375 (TR_34_) and 0.0666 (TR_46_); Fig. 7A, middle]. The observed weight gain in wild-type-infected posaconazole-treated mice was accompanied by a significantly increased survival (2/3) at day 6 (endpoint) compared to triazole-resistant inoculated mouse groups (Fig. 7B, middle) for which none of the infected mice had survived (0/3 per group) by the end of the experiment [*P*=0.0485 (TR_34_), 0.2313 (TR_46_)]. TRAF mice presented with significantly increased bioluminescence signals [*P*=0.0378 (TR_34_) and 0.0383 (TR_46_); Fig. 7C,D, middle) and increased lung lesion development [non-aerated volume, *P*=0.1923 (TR_34_) and 0.1833 (TR_46_), and clinical score *P*=0.0352 (TR_34_) and 0.0538 (TR_46_), respectively; Fig. S9A-C, middle] compared to WT-infected mice at day 3. CFU counts were likewise increased in the triazole-resistant infected groups compared to wild-type-infected mice [*P*=0.0127 (TR_34_) and 0.0021 (TR_46_); Fig. 7E, middle]. Accordingly, treatment with posaconazole had a beneficial effect on the outcome and disease development only in mice infected with our susceptible strain. On the contrary, no beneficial effect on disease development or fungal burden reduction was observed among posaconazole-treated mice infected with either of our triazole-resistant strains, with comparable effects to those reported in the TR_34_- and TR_46_-infected placebo-treated groups (Fig. 7E, left) on survival [*P*=0.8864 (TR_34_), 0.3701 (TR_46_)], weight loss [*P*=0.4229 (TR_34_), 0.0700 (TR_46_)], fungal burden [BLI signals, *P*=0.7126 (TR_34_), 0.2890 (TR_46_)], CFU counts [*P*=0.6624 (TR_34_), >0.9999 (TR_46_)] and lung lesion development [*P*=0.4145 (TR_34_), 0.3041 (TR_46_)]. Thus, the observed therapeutic failure of posaconazole treatment is directly associated with the triazole-resistant phenotype of the triazole-resistant strains.

Treatment with L-AmB of susceptible and TRAF-infected groups increased survival in most mice (2/3, 67%) from each infected group (Fig. 7B, right) at the end of the experiment (day 6) compared to none (0/3) in the placebo groups and none (0/3) in the posaconazole-treated mice infected with triazole-resistant strains (*P*=0.0253; Fig. 7B, left and middle). An initial transitional increase in weight loss, bioluminescence signals and lung lesion development was observed among L-AmB-treated mice compared to baseline measurements until days 2 or 3 after infection (Fig. 7A,C-E; Fig. S9, right). These increases were followed by a subsequent gradual recovery of weight and a decrease in bioluminescence emission and lung lesions, with no significant differences between susceptible and triazole-resistant infected groups at day 6 [*P*=0.9999 (TR_34_) and *P*≥0.9999 (TR_46_)]. Likewise, CFU counts were significantly decreased in all L-AmB-treated mice compared to placebo-treated mice [*P*=0.0415 (wild type), *P*=0.0500 (TR_34_) and *P*=0.0305 (TR_46_); Fig. 7E, left and right) at endpoint, inferring that the observed positive effects of L-AmB treatment on survival and disease development are a result of decreased fungal burden in the lungs.

Overall, our results demonstrate that our newly developed red luciferase-expressing *A. fumigatus* strains are suitable for drug treatment efficacy studies for IPA, not only in susceptible infections but also in triazole-resistant scenarios, as implied by the observed therapeutic failure after posaconazole treatment in triazole-resistant infected mice and the beneficial effects of L-AmB in all infected mice groups, enabling the tracking of fungal burden (BLI) as early as from the first day of infection.

## DISCUSSION

In this study, we established and optimized, using a stepwise approach, a multimodal imaging-compatible reproducible neutropenic mouse model of IPA, with increased fungal burden detection using newly engineered red-shifted luciferase expressing *A. fumigatus* strains to study triazole-susceptible and triazole-resistant infection scenarios and their treatment.

Red-shifted firefly luciferases (600-700 nm emission) are known to have less light scattering and absorption in tissues compared to luciferases emitting in the ‘green’ spectrum, and have been successfully implemented to study superficial and deep-seated infections in microorganisms, such as *C. albicans* and *C. neoformans* (yeasts) but not in filamentous fungi, such as *A. fumigatus* (Vanherp et al., 2019; Dorsaz et al., 2017; Avci et al., 2018). We successfully engineered a codon-optimized red-shifted thermostable firefly luciferase and introduced it into the *akuB* locus of an *A. fumigatus* reference strain under constitutive luciferase expression from the *gpdA* promoter. As the accompanied deletion of the *akuB* results in increases in the frequency of homologous integration without loss of pathogenicity in murine models (Da Silva Ferreira et al., 2006), we used this novel triazole-susceptible bioluminescent strain to generate isogenic *A. fumigatus* triazole-resistant strains by replacing the wild-type *cyp51A* gene with the most commonly reported *cyp51A* gene mutations conferring triazole-resistance, which are the TR_34_/L98H and the TR_46_/Y121F/T289A mutations (Resendiz Sharpe et al., 2018; Nywening et al., 2020). We specifically aimed to generate triazole-susceptible and triazole-resistant isogenic strains to exclude effects from other strain variations when aiming for comparative studies between our strains in murine model systems of infection. The introduced *cyp51A* mutations caused the expected triazole resistance, whereas all other tested phenotypic characteristics remained unchanged to the parental bioluminescent strain. Therefore, these strains will be invaluable when directly comparing triazole or combination therapies in triazole-sensitive and triazole-resistant settings. It is important to note that as in all genetically modified microorganisms, the genetic modifications introduced in our novel strains, such as the pyrithiamine resistance marker, could potentially create confounding issues in specific settings, such as in drug treatment studies involving thiamine biosynthesis. The observed results may not always be representative to other *A. fumigatus* strains.

As expected, the red-shifted luciferase from all strains produced peak total flux emissions in the red spectrum (620 nm) and caused no repercussions in the fungal cell physiology, as determined by sporulation efficiencies, spore viability and growth kinetics that were comparable to the parental reference strain. Furthermore, *in vitro* bioluminescence emissions from our newly developed strains matched those of the Af2/7/1 strain expressing a luciferase in the green spectrum, as light scattering secondary to adjacent tissue-light absorption is absent under *in vitro* conditions. However, although the bioluminescent Af2/7/1 *A. fumigatus* strain has been successfully used to detect deep-seated *Aspergillus* tissue infections in neutropenic mouse models *in vivo* (Poelmans et al., 2018; Galiger et al., 2013), bioluminescence signals were only detected at significant levels in later stages of the disease (Poelmans et al., 2018; Ibrahim-Granet et al., 2010). This limited the number of data points and relevant information on disease dynamics at the early stages of infection. By contrast, we demonstrated that BLI signals from the red-shifted luciferase-expressing strains engineered in this study have increased *in vivo* light emission capabilities in tissues, as confirmed by the detection of higher total flux values compared to the Af2/7/1 strain. The observed BLI proficiencies of the red-shifted strains improved the sensitivity of fungal burden detection in our neutropenic model, allowing us to follow up disease development as early as day 1 after infection, which is 2 days earlier than in most previous studies and enables crucial information on disease onset and progression to be obtained. The increased *in vivo* BLI signals of our strains can be solely attributable to their red spectrum of light emission as all tested strains presented comparable lung disease development, as assessed by micro-CT and fungal burdens quantified by CFU counts.

To investigate IPA development *in vivo*, it is essential to apply an immunosuppressive regimen that renders all mice neutropenic and confers the least compromise to the overall health of the animals (<15% weight loss). However, among the limited *in vivo* murine studies that have monitored the effects of cyclophosphamide-dose regimens on neutrophil population kinetics, variable dosing regimens between 500 mg/kg and 100 mg/kg have been used with variable outcomes. Moreover, none determined the severity of the effects of these regimes on the overall health of the mice (weight loss or survival) (Desoubeaux and Cray, 2017; Brunck et al., 2014; Zuluaga et al., 2006). Hence, in our study, we compared several immunosuppressive regimens to determine the most suitable drug administration.

A cyclophosphamide regimen of 150 mg/kg administered i.p. at day −4 and −1 rendered all mice neutropenic for ∼3 days, as reported by Brunck et al. (2014), and this was prolonged by a booster dose on day 2 for a total of 5 days consecutively (endpoint). We did not see this effect in the 100-mg/kg dosing regimen, as our results showed neutropenia until day 2 despite a boosting dose on day 3, whereas Brunck et al. (2014) showed a more extended period of neutropenia in female mice, but the reasons for this are not clear. This discrepancy is unlikely to be due to the broader age range (6-10 weeks) of the animals used by Brunck et al. (2014), as it includes our age-range (8-10 weeks). Additionally, we can dismiss gender effects when comparing the described 150-mg/kg dose regimen with a booster at day 2 in both male and female mice, as we observed delayed immune recovery for several days regardless of the sex of the animal. For these reasons, our regimen was identified as the most suitable for studying disease progression and treatment responses in neutropenic scenarios. Besides excluding potential gender-related differences in response to immune suppression, we also corroborated that IPA develops equally in male and female cyclophosphamide-immunosuppressed infected mice. This enables researchers to include male and female mice in experiments to overcome issues such as gender bias.

Based on our findings, the potential concomitant use of grapefruit juice in voriconazole therapy (Sugar and Liu, 2000) upon *A. fumigatus* infection in cyclophosphamide-immunosuppressed mice should be avoided as the health of mice, based on weight loss and survival, was severely compromised in all tested cyclophosphamide regimens; alternatives should be further investigated. Other triazoles, such as posaconazole therapy, which is also recommended for prophylaxis and therapy of IPA (Tissot et al., 2017; Patterson et al., 2016), can be used without grapefruit juice as it has increased bioavailability in mice (Seyedmousavi et al., 2015). However, posaconazole drug monitoring is warranted as the threshold (MICs) between resistance and susceptibility are much lower than those observed for other triazoles, as confirmed by triazole resistance analyses of our strains (Table 1).

A reproducible lung infection is crucial, especially when aiming to keep the number of animals in individual groups small. The intranasal route of infection is widely used in murine models of infection, as it allows an easy administration of the inoculum (Desoubeaux and Cray, 2017). However, differences in the fungal load deposited in the lungs have been reported (Desoubeaux and Cray, 2017), whereby in some cases only 10% of the initial inoculum reached the lung, with most spores remaining in the upper respiratory tract. This problem was also evidenced by high sinus BLI signals in some of our intranasally infected mice (Af_luc_OPTred__WT). Therefore, we propose an orotracheal route of inoculation. Although inoculum application by this route requires more training, results demonstrated that it favored a more efficient conidia deposition in the lower respiratory tract and more homogenous IPA development, and, as such, allowed the reduction of mice in individual groups. Compared to the intranasal route of infection, the orotracheal route significantly increased lung lesion development (micro-CT scans) and fungal lung burden (BLI signal and CFU counts), confirming that this method is a more suitable route of inoculation for IPA studies.

In our study, the *in vivo* imaging biomarker readouts given by bioluminescence and micro-CT provided a good correlation with the fungal load obtained in the lungs of infected mice. Good correlation of CFUs with BLI and micro-CT, and of bioluminescence signals with kidney CFUs, have previously been reported in a mouse model of Cryptococcosis (Vanherp et al., 2019) and in systemic *C. albicans* infections, respectively (Jacobsen et al., 2014). Hence, as imaging techniques have several advantages to single-endpoint readout techniques, such as CFU counts that require an increased number of animals and have variable reproducibility, lung imaging biomarkers are powerful tools for studying IPA as they provide crucial day-to-day information at early stages of disease development in a single infected mouse, and fungal burden kinetics from each mouse can be presumed and followed up longitudinally. Moreover, longitudinal imaging overcomes residual inherent inter-animal variability and increases experimental power due to an increased number of timepoints with a lower number of animals, which is of particular interest in treatment studies and for further reducing experimental complexity and variability.

Although we observed a generally improved detection of the fungal burden by using a red-shifted luciferase in combination with the standard administered luciferin dose of 126 mg/kg, the signals at the onset of infection remained low. Therefore, we tested whether the sensitivity of BLI in *A. fumigatus* could be further improved by increasing the bioavailability of luciferin in lung tissues by increasing the luciferin concentration in the i.p. injections (Berger et al., 2008). An excess of luciferin is required for maximum light output, and a possible explanation for lower signals from fungal cells may derive from the complexity of the cell wall of *A. fumigatus* hyphae, which might delay or restrict luciferin uptake. Another limitation could involve the co-substrate adenosine triphosphate (ATP), which is required for the optimal function of firefly luciferases (Papon et al., 2014). The generation of cellular ATP might differ in fungi compared to other cell types and subsequently the luciferase reaction (Brock, 2012; Calderone et al., 2015). We observed that increasing the luciferin dose from 126 mg/kg to 250 mg/kg, and further to 500 mg/kg, exponentially increased the sensitivity of BLI signal detection (total flux) in a dose-dependent manner, with no delay in time-to-peak or saturation in infected mice (Af_luc_OPT_red__WT). The effects of increased luciferin substrate concentration on *in vivo* bioluminescence production by diverse luciferases have been described in other microorganisms, such as *Mycobacterium smegmatis* and mammalian cells (pancreatic, brain and immune cells) (Andreu et al., 2010, 2013; Aswendt et al., 2013; Chen and Kaufman, 2009). Andreu et al. (2013) detected an exponential total flux increase in a model of *M. smegmatis* infection as luciferin doses increased from 300 mg/kg to 500 mg/kg. In our study, the luciferin dose of 500 mg/kg resulted in the most significant increase in BLI signal, with sensitive detection of infection as early as day 1 post-infection, with no indications of toxicity. The dose-dependent increase in BLI signal and luciferin substrate concentrations was further confirmed by significantly increased BLI signals in the 126-mg/kg and 250-mg/kg dose groups after topping the dose up to a total concentration of 500 mg/kg. However, the topped-up BLI signals did not reach the levels observed in the 500-mg/kg group and were significantly lower, suggesting a higher absorption of luciferin by the intestinal track from two subsequently injected doses compared to a single high dose (Berger et al., 2008). Dose-dependent BLI signal increase in luciferin doses up to 750 mg/kg have been reported in mammalian cell neuroimaging studies without signs of toxicity, which could be further investigated in our model (Aswendt et al., 2013). Increasing the luciferin concentration is of particular interest in scenarios with low fungal burdens, such as early stages of disease development and antifungal drug efficacy studies, or in scenarios in which the availability of luciferin is restricted due to poorly vascularized regions, such as in necrotic tissue.

Lastly, we successfully tested and validated the use of our multimodal murine imaging model of IPA and newly developed triazole-susceptible and resistant bioluminescent strains for therapeutic efficacy studies in both triazole-susceptible and -resistant settings. Weight loss, survival and imaging biomarkers consistently, sensitively and significantly enabled us to detect longitudinal variations in fungal burdens and lung lesion formations between different antifungal therapies (posaconazole, L-AmB and placebo) in triazole-susceptible and -resistant *A. fumigatus* infections. *In vivo* and clinical studies have reported that IPA caused by TRAF is more likely to have no therapeutic response to triazole antifungal therapy with fatal outcomes (Resendiz Sharpe et al., 2018; Resendiz-Sharpe et al., 2019; Lepak et al., 2013). In our model, posaconazole treatment among mice infected with either of the triazole-resistant strains likewise presented therapeutic failure, and was characterized by significantly increasing fungal burdens and lung lesion development over time, with comparable clinical deterioration (survival and weight loss) to mice receiving the placebo treatment.

Interestingly, even though all posaconazole-treated mice infected with either the triazole-resistant Af_luc_OPT_redt__TR34 or _TR46 strains succumbed at the end due to therapeutic failure, a trend of prolonged survival between day 3 and day 6 in both infected groups, and a trend of decreased CFU counts in the TR34 infected group, was observed compared to placebo-treated mice infected with the same triazole-resistant strains. Although these observed differences did not achieve significance, they indicate that triazole antifungals may still improve infection outcome even in TRAF infections, albeit to a limited extent. The susceptibility of triazole antifungals *in vitro* cannot fully predict how these drugs will perform in the *in vivo* context (Rosowski et al., 2020; Mavridou et al., 2010), as therapeutic responses may depend on many factors. For example, the degree of immunosuppression is known to be a significant driver in IPA development (Kosmidis and Denning, 2015). In some scenarios, the remaining immune cellular response might be enough to partially control or delay fungal growth. In addition, isolated epithelial cells and murine macrophages can take up and concentrate the triazole posaconazole *in vitro* (Rosowski et al., 2020; Campoli et al., 2013). This may also be the case in animal models in which increased drug availability could temporarily reach sufficiently high concentration levels to inhibit the growth of TRAF spores (Rosowski et al., 2020; Campoli et al., 2013). It has also been shown that the triazole voriconazole can independently protect against *A. fumigatus* infection, even in triazole-resistant scenarios, through direct interaction with the host innate immune cells, such as macrophages and neutrophils (Rosowski et al., 2020; Simitsopoulou et al., 2007; Vora et al., 1998). These antifungals induced a more pronounced immune gene expression response in the presence of hyphal fragments *in vitro*, potentially leading to more efficient host resistance to *A. fumigatus* (Simitsopoulou et al., 2007). ﻿The effect of triazole antifungals on delaying disease development and host-immune interactions in triazole-resistant infections is an essential aspect of future study that could be further elucidated with our model.

Concerning L-AmB treatment, we observed beneficial antifungal therapeutic effects in infected mice with triazole-resistant strains and our susceptible strains, which were all sensitive to L-AmB *in vitro*. Thus, our experiments proved suitable for *in vivo* therapeutic assessment monitoring and confirmed L-AmB as a treatment option in infections with triazole-resistant strains. Furthermore, the strains and methods developed and refined in this study offer the research community a much needed tool to investigate IPA infections in triazole-susceptible and -resistant settings. Besides enabling the detection of the fungal burden (BLI) from as early as the first day of infection, unlike previous therapeutic assessment studies (Poelmans et al., 2018; Galiger et al., 2013), our isogenic bioluminescent TRAF strains provide the unique opportunity to use BLI to longitudinally follow up the kinetics of IPA development in triazole-resistant infected mouse from the onset of antifungal therapy (novel antifungal drugs or rediscovery of old drugs), offering crucial daily information about therapeutic effects on the fungal burden (bioluminescence) and disease development within the same animal.

In conclusion, we developed an optimized reproducible neutropenic murine model of IPA, with accurate pulmonary infection by the orotracheal route accompanied by increased fungal burden detection sensitivity through the generation of triazole-susceptible and -resistant *A. fumigatus* strains expressing a red-shifted luciferase, and boosted luciferin dosage. Our findings provide a unique framework for studying IPA, from early stages of disease development down to different therapeutic settings. As reports of triazole resistance in *A. fumigatus* increase worldwide, the generation of two bioluminescent strains resistant to triazole antifungals, harboring the two most commonly reported distinctive genetic mechanisms of resistance in *A. fumigatus*, provides unique tools for studying differences in disease development, host-pathogen interactions and therapeutic approaches for triazole susceptible, triazole resistant and mixed infections.

## MATERIALS AND METHODS

### Construction of wild-type *A. fumigatus* strains expressing a red-shifted thermostable firefly luciferase

To generate bioluminescent *A. fumigatus* strains with red spectrum light emission, we synthesized a codon-optimized red-shifted thermostable firefly luciferase using a codon-optimized wild-type luciferase (accession number KC677695) as template and introducing the mutations S284T, F295L and E354K (lucOPT__red_TS_, GenScript; accession number MT554554); as described for the monitoring of infections caused by *C. neoformans* (Vanherp et al., 2019). All PCR reactions for generating the individual constructs were performed using Phusion polymerase (Fisher Scientific, USA) and oligonucleotides described in Table S2. An 820-bp promoter of the *Aspergillus nidulans* P*gpdA* and a 322-bp *gpdA* terminator region (T*gpdA*) were amplified from genomic DNA of the *A. nidulans* FGSC A4 wild-type strain. PCR fragments were fused up and downstream of the luciferase to generate the luciferase reporter. The construct was cloned into the *ptrA*-pJET1 plasmid containing the pyrithiamine resistance gene (Fleck and Brock, 2010). This reporter/resistance cassette was amplified by PCR using oligonucleotides PgpdAIFKu80up_f and ptrAIFKU80do_r. Subsequently, the upstream (819 bp) and downstream (666 bp) regions of the *A. fumigatus akuB* gene were amplified using genomic DNA of the *A. fumigatus* wild-type strain CBS144.89 as template (upstream region primers, KU80upIFpUC_f and KU80upIFPgpdA_r; downstream-region, KU80doIFptrA_f and KU80doIFpUC_r). All three PCR products were assembled by *in vitro* recombination in a SmaI-restricted pUC19 plasmid using an InFusion HD cloning kit (Takara/Clonetech, Japan) and amplified in *Escherichia coli* DH5α. Plasmid DNA was isolated using a NucleoSpin plasmid kit according to the manufacturer's instructions (Machery-Nagel, Germany). The Δ*akuB::luc*_OPT_red__*ptrA* cassette (Fig. S1A) was excised from the plasmid backbone through SmaI restriction and used for protoplast transformation of the *A. fumigatus* wild-type strain CBS144.89 using 0.1 μg pyrithiamine/ml (Sigma-Aldrich, UK) as a selection marker. Transformants were cultivated on *Aspergillus* minimal medium containing 0.2 mM D-luciferin (Promega) and pre-screened by imaging (chemiluminescence setting) using a Chemi-Doc XRS system (Bio-Rad, USA). Selected transformants were analyzed for single-copy integration of the reporter construct into the *akuB* locus by Southern blotting using a digoxygenin-dUTP labeled probe against the *akuB* downstream region (Fig. S2A). The strain Af_luc_OPT_red__WT (Af_Δ*akuB::luc*_OPT_red__ptrA No. 5) was selected for further *in vitro* and *in vivo* characterization, and served as parental strain for generating the isogenic triazole-resistant strains.

### Generation of triazole-resistant *A. fumigatus* strains with red-shifted thermostable luciferase expression

To develop isogenic *A. fumigatus* triazole-resistant strains, we substituted the wild-type *cyp51A* gene of the Af_*luc*_OPT_red__WT *A. fumigatus* strain with a *cyp51A* gene version harboring either the TR_34_/L98H or the TR_46_/Y121F/T289A mutation. Briefly, the promoter sequence of the *cyp51A* gene, containing the tandem repeat and a part of the coding region comprising the point mutations of the respective *cyp51A* gene, was amplified from the clinical isolates V-052-35 (TR_34_/L98H) and CYP-15-7 (TR_46_/Y121F/T289A) using the oligonucleotides pCYP51A_f and tCYP51A_r. The PCR products were gel-purified and directly used for PEG-mediated transformation of the Af_luc_OPT_red__WT strain. The transformants were selected by the addition of 4 µg/ml of itraconazole to the transformation medium (GG10 with 1.2 M sorbitol) (Geib and Brock, 2017). The presence of a single-copy integration of the partial *cyp51A* gene into the *cyp51A* locus was confirmed by Southern blotting (Fig. S2B). The resistant phenotype of selected transformants was determined and confirmed using the European Committee on Antimicrobial Susceptibility Testing (EUCAST) broth microdilution reference methodology for filamentous fungi and clinical breakpoints for *A. fumigatus* (MIC: itraconazole >1 mg/l, posaconazole >0.25 mg/l and voriconazole >1 mg/l) (The European Committee on Antimicrobial Susceptibility Testing, www.eucast.org/astoffungi/clinicalbreakpointsforantifungals/; EUCAST clinical breakpoints for fungi, http://www.eucast.org.http//www.eucast.org). Mutations in the *cyp51A* gene were confirmed by sequencing as described previously (Vermeulen et al., 2015). The transformants Af_luc_OPT_red__TR46 (Af_Δ*akuB::luc*_OPT_red_TS__*ptrA*_4003-new7) harboring the TR_46_/Y121F/T289A *cyp51A* gene mutation and the Af_luc_OPT_red__TR34 (Af_Δ*akuB::luc*_OPT_red_TS__*ptrA*_3216-1) harboring the TR_34_/L98H were selected for further analysis.

To corroborate that the observed resistance phenotype in our transformants was conferred by the introduced mutations in the *cyp51A* gene, we replaced their mutated *cyp51A* gene with the wild-type *cyp51A* gene (CBS144.89). Briefly, the oligonucleotides CypAfHind_up_f and CypAfNotTer_r were used for amplifying a fragment containing the promoter region (986 bp), CDS (1619 bp) and terminator region (228 bp) of the wild-type *cyp51A* gene. A 792-bp fragment of the downstream region was also amplified using the CypAfNotDown_f and CypAfHindDown_r oligonucleotides. The two fragments were assembled by *in vitro* recombination in a HindIII-digested pUC19 plasmid, and the construct was amplified in *E. coli* DH5α. The hygromycin B resistance marker was cloned into the complementation construct using a NotI site introduced between the two downstream fragments. To perform the transformation of Af_Δ*akuB::luc*_OPT_red__*ptrA*_4003-new7 and Af_*ΔakuB::luc*_OPT_red__*ptrA*_3216-1, the complementation construct (Fig. S1B) was released from the vector backbone by HindIII restriction and then gel-purified. Selection of transformants was performed using hygromycin B (180 µg/ml) as a selection marker. Single-copy integration into the *cyp51A* locus was verified by Southern blotting (Fig. S2C), and restoration of the wild-type sequence by *cyp51A* gene sequencing. The sensitivity of the complemented strains Af_luc_OPT_red__TR34_comp_3216-1_No.23_hygR and Af_luc_OPT_red__TR46_comp_4003 No.15_hygR against triazole antifungals (voriconazole, posaconazole, itraconazole; Sigma-Aldrich, USA) was tested using the EUCAST broth microdilution methodology.

### *Aspergillus fumigatus* strains

For experimental assays, stock suspensions from each strain (1×10^8^ spores/ml) were made and stored for further experiments. Briefly, strains were cultured for 3 days at 37°C on Sabouraud agar tubes and harvested by adding 5 ml of distilled water-0.1% Tween 80 (Sigma-Aldrich, USA) and gently scraping off colonies from the surface with a disposable loop. The collected suspension was vigorously vortexed and filtered (syringe filter holder with an 11-μm nylon net filter; MerckMillipore, Ireland) to remove hyphae or spore clumps. Spore suspensions were subsequently centrifuged, washed and reconstituted in saline solution for Tween 80 removal. Using a Neubauer hemocytometer, spores were counted, aliquoted and stored (−80°C). For experiments, conidia were thawed, enumerated and diluted based on required experimental setting amounts (Ito et al., 2006); spore viability was assessed by agar plating.

### Sporulation efficiency assay

For each tested strain, three small tissue culture flasks containing Sabouraud agar (25 cm^2^ treated, vented cap, sterile; VWR International, USA) were inoculated with 4×10^6^ spores per flask (McCormick et al., 2012). After incubation for 4 days at 37°C, conidia from each culture flask were harvested by adding 10 ml of distilled water-0.1% Tween 80 (Sigma-Aldrich, USA), filtering (11 μm pore diameter) and counting using a Neubauer chamber. Average sporulation counts per strain group (*n*=3) were determined. Statistical significance was determined by one-way ANOVA with multiple comparison analysis with Bonferroni correction.

### Spore viability assay

Spore viability was determined as the ratio from an initial spore inoculum (1×10^8^ resting conidia) and subsequent CFU counts (10-fold series dilution) after incubation for 48 h at 37°C. A ratio of 1 was considered as 100% viability (initial inoculum and cultured CFU counts relationship). This experiment was performed in triplicate and significance was calculated by one-way ANOVA with multiple comparison analysis with Bonferroni correction.

### Growth curves

Growth of the genetically modified red-shifted luciferase-expressing strains and of the Af2/7/1 (codon-optimized wild-type luciferase, ‘green’ spectrum; Galiger et al., 2013), V-052-35 (TR_34_/L98H) and CYP-15-7 (TR_46_/Y121F/T289A) equivalents were determined and compared to the wild-type CBS 144.89 strain. Briefly, a 100-µl suspension containing 2.5×10^5^ spores was added to 100 µl of 2× RPMI 1640 (Sigma-Aldrich, UK) with 2% glucose medium in 96-well tissue-treated microdilution plates (NunclonTM Delta-surface, Thermo Fisher Scientific, Denmark) (The European Committee on Antimicrobial Susceptibility Testing) Microdilution plates were incubated without agitation at 37°C for 24 h. Optical density (OD)_405_ nm was measured at 0, 1, 2, 3, 4, 5, 6, 7, 8, 9, 10, 11, 22 and 24 h using a Wallac Victor 1420 multilabel counter reader (PerkinElmer, USA). Sequential OD measurements were used to generate growth curves for each strain (8 wells per strain). Simple linear regression analysis and growth curves correlation (Pearson r) to the CBS 144.89 wild-type strain were determined and used as estimates of the growth rates of each strain analyzed.

### *In vitro* bioluminescence measurements

To confirm the bioluminescence emission capabilities of red-shifted bioluminescent strains, serial 4-fold dilutions of fungal inoculums (1×10^7^ initial spore count) resuspended in 200 µl of PBS and D-luciferin (final concentration 0.15 µg/ml, Luciferin-EF, Promega, USA) were transferred to a 96-well plate (Nunclon Delta-surface, Thermo Fisher Scientific, Denmark); a well without conidia (PBS+luciferin) was used as a control. BLI signal in samples was measured using an IVIS Spectrum imaging system (PerkinElmer, USA). Consecutive images were acquired for 10 min using an exposure time of 30 s (open filter, F/stop 1, subject height of 0.5 cm and medium binning). Peak total flux was quantified using Living Image Software (version 4.5.4) from a circular region of interest (ROI) of 0.8 cm diameter covering each well. Red-emission spectra (total flux) characteristics of the red-shifted luciferase were determined using a range of emission filters from 520 to 740 nm, and compared to the BL Af2/7/1 strain (green spectrum). The LOD of ungerminated conidia of each bioluminescent strain was determined as the last significant measurement (total flux) above background levels of the serial 4-fold dilutions of fungal inoculums (1×10^7^ initial spore count) compared to control well measurements.

### Animals and model induction

In all our *in vivo* experiments, 8-10-week-old BALB/cAnNCrl mice (Charles River Laboratories, France) were housed in individually ventilated cages with free access to food and water. To reduce the risk of bacterial infection, an antibiotic (Baytril – Enrofloxacin 50 mg/kg/day, Bayer, Belgium) was added to the drinking water at the initiation of the immunosuppressive regimen. Animals were randomly assigned to experimental groups. Mice were monitored daily for body weight, general condition and presence of respiratory distress during experiments, until a predefined experimental or humane endpoint was reached (>20% weight loss, lethargy and/or respiratory distress). All animal experiments were approved by the animal ethics committee of KU Leuven (ECD project P227/2018) in accordance with national and European regulations.

### Immunosuppression regimens *in vivo*

Mice were rendered neutropenic by i.p. injections of cyclophosphamide (0.9% saline solution; Sigma-Aldrich, Belgium) according to pre-assigned immunosuppressive regimens. The following regimens, in which day 0 represents the day of intended inoculation, were used: (A) 150 mg/kg on days −4 and −1; (B) 150 mg/kg on days −4 and −1 with a booster (additional dose) of 150-mg/kg dose on day 2; (C) 100 mg/kg on days −4, −3, −2 and −1; and (D) 100 mg/kg on days −4, −3, −2, and −1 with a booster of 100 mg/kg dose on day 3. In addition, grapefruit juice was used as a substitute for water (initiated at day −2; Carrefour commercial brand) to determine its effects on immunosuppression in two groups based on the regimen doses A (regimens E) and dose C (regimens F). Each regimen consisted of three mice per timepoint (five timepoints; day 0 to day 4). Mice were monitored daily for weight loss and survival. Blood was drawn by cardiac puncture and anticoagulated using 3.8% trisodium citrate (1 unit per 9 parts of blood) for analysis. Blood cells counts were measured at predefined timepoints using an Advia 2120i hematology system (Siemens Healthcare GmbH, Germany). Severe neutropenia was considered as <100 neutrophils/µl.

### Antifungal drugs

The human clinical oral formulation of posaconazole (Noxafil suspension; Merck Sharp & Dohme, USA) was used in this study. For administration, a 10-fold dilution was performed using sterile water to achieve a concentration of 4 mg/l (initial concentration 40 mg/l) to reach a concentration of 8 mg/kg for oral gavage administration. L-AmB was prepared as described previously (Galiger et al., 2013). Briefly, lyophilized L-AmB (AmBisome; Gilead Sciences, USA) was reconstituted in sterile water to a final concentration of 5 mg/ml. The obtained solution was further diluted in sterile 5% dextrose to a final concentration of 2.5 mg/l to reach a concentration of 10 mg/kg dose per mouse to be i.p. administered.

### Mouse model of invasive pulmonary aspergillosis

Groups of five 8-9-week-old mice were immunosuppressed (150 mg/kg cyclophosphamide dose; day −4, −1+booster day 2) and inoculated with 20 µl of fungal inoculum suspension (5×10^5^ spores in PBS) of the corresponding strain through either an intranasal or orotracheal (intratracheal) route as described previously (Revelli et al., 2012). The 5×10^5^ spores dose was chosen for inoculation as variable infection rates have been associated with lower inoculation doses in mice (Poelmans et al., 2018). Anesthesia induction for inoculation was performed by either gas anesthesia with 2% isoflurane (Piramal Healthcare, UK) in 100% O_2_ (nasal cone), or by i.p. injection with ketamine and medetomidine followed by atipamezole reversal (Cruz et al., 1998). The day of fungal inoculation was considered as experimental day 0. Sacrifice was performed using an overdose of pentobarbital via i.p. injection.

For treatment experiments, therapy was initiated 1 h after infection. The triazole posaconazole was administered daily via oral gavage (8 mg/kg dose). L-AmB was administered every day by i.p. injections according to stated concentration. Placebo control mice were likewise treated daily by i.p. injections of saline solution (50 µl). As the posaconazole threshold (MICs) between resistance and susceptibility is much lower than those observed for other triazoles (Table 1), drug monitoring of posaconazole in serum was performed in mice treated with this antifungal at sacrifice day using ultra-performance liquid chromatography-tandem mass spectrometry to ensure adequate therapeutic antifungal concentrations. For *A. fumigatus* isolates considered as susceptible to posaconazole (MIC, <0.25 mg/l^55^), a posaconazole serum concentration between 1.2 to 2.9 mg/l at 24 h was determined as target (Seyedmousavi et al., 2014; The European Committee on Antimicrobial Susceptibility Testing, www.eucast.org/fileadmin/src/media/PDFs/EUCAST_files/AFST/Files/EUCAST_E_Def_9.3.2_Mould_testing_definitive_revised_2020.pdf).

### *In vivo* bioluminescence imaging

Bioluminescence images were acquired daily (baseline day −1 and from day 1 onwards) using an IVIS Spectrum system (PerkinElmer, USA). Animals first received an i.p. injection with D-luciferin (126 mg/kg standard dose experiments; 250 mg/kg or a maximum of 500 mg/kg for the increased dosing experiment). Mice were subsequently anesthetized with gas anesthesia as described previously and placed in a heated flow chamber in a supine position for image acquisition (Poelmans et al., 2018). Acquisition of consecutive frames (10-15 frames of 60 s each, f/stop 1, medium binning, height 1.5 cm) was started until maximum signal intensity was reached (peak signal intensity, ∼25 min after injection), measured as photon flux per second through a rectangular ROI covering the lung area (2.1 cm width×2.6 cm height) or sinus area (1.2 cm width×1.5 cm height) using Living Image Software (version 4.5.4, PerkinElmer, USA).

### Micro-computed tomography

Micro-CT data were acquired using a small animal micro-CT scanner (SkyScan 1278, Bruker micro-CT, Belgium) and the following scan parameters: 50 kV X-ray source, 1 mm aluminum X-ray filter, 350 µA current, isotropic reconstructed voxel size 50 µm and 150 ms exposure time per projection with 0.9° increments, resulting in a total scanning time of ∼3 min (Berghen et al., 2019). For image acquisition, mice were anesthetized using gas anesthesia and placed in a heated chamber in a supine position. Micro-CT images were acquired daily (baseline day −1 and from day 1 onwards). Respiratory weighted micro-CT-data were reconstructed, visualized and processed using NRecon (version 1.6.10.4), Data Viewer (version 1.5.2.4) and CTan (version 1.16.3.0) software provided by the manufacturer. The following parameters were used for image reconstruction: smoothing 1 and beam hardening correction of 10%; ring artefact reduction and post alignment were adjusted per scan. For image analysis, a volume of interest covering both lungs entirely was manually delineated avoiding the heart and main blood vessels on transverse images for each animal at each timepoint. Micro-CT derived biomarkers of lung pathology (total lung volume, aerated lung volume, non-aerated lung volume and mean density of total lung volume) were subsequently quantified as described previously (Berghen et al., 2019). For micro-CT clinical score determination, visual observations of coronal images were semi-quantitatively scored using four images at predefined positions in the lung for each animal at each timepoint. At baseline scans, a score of zero was given to each image; later scores based on the increase (+0.25), stabilization/no change (0) or improvement (−0.25) of pulmonary lesions were assigned each day to obtain a cumulative score (Vande Velde et al., 2016). Micro-CT analysis were performed separately by two researchers and the results averaged.

### Lung colony-forming unit analysis

For CFU counts determination, lungs were homogenized in 600 µl saline solution. A 10-fold dilution series (total volume 1000 µl) was prepared from homogenized suspension and plated (100 µl) on Sabouraud agar plates (plus chloramphenicol). Plates were incubated at 37°C and counted at 48 h. CFU counts were expressed in log^10^ CFU counts per gram of lung tissue.

### Statistical analysis

All statistical analyses were performed using GraphPad Prism version 9.0.1. (GraphPad Software, USA). Survival analysis was performed using the log rank (Mantel–Cox) test. Simple linear regression analysis with Pearson correlation analysis was used for growth curves evaluation. Repeated measurement ANOVA testing with multiple correction comparison were performed for statistical hypothesis testing between groups. Statistical analyses were performed with a two-sided alternative hypothesis at the 5% significance level. In graphs, significance (*P*-value) indication is depicted above the graph when comparing timepoint per group to its own baseline. Significance was depicted next to the graph (right side) when comparing multiple groups over time in a figure.

## Supplementary Material

Supplementary information
