# Supplementary material for: Longitudinal multimodal imaging-compatible mouse model of triazole-sensitive and -resistant invasive pulmonary aspergillosis
Source: Dis Model Mech. 2022 Mar 30;15(3):dmm049165. doi: 10.1242/dmm.049165 (PMC8990085; doi:10.1242/dmm.049165)
Supplement: Supplementary information [file dmm-15-049165-s1.pdf]

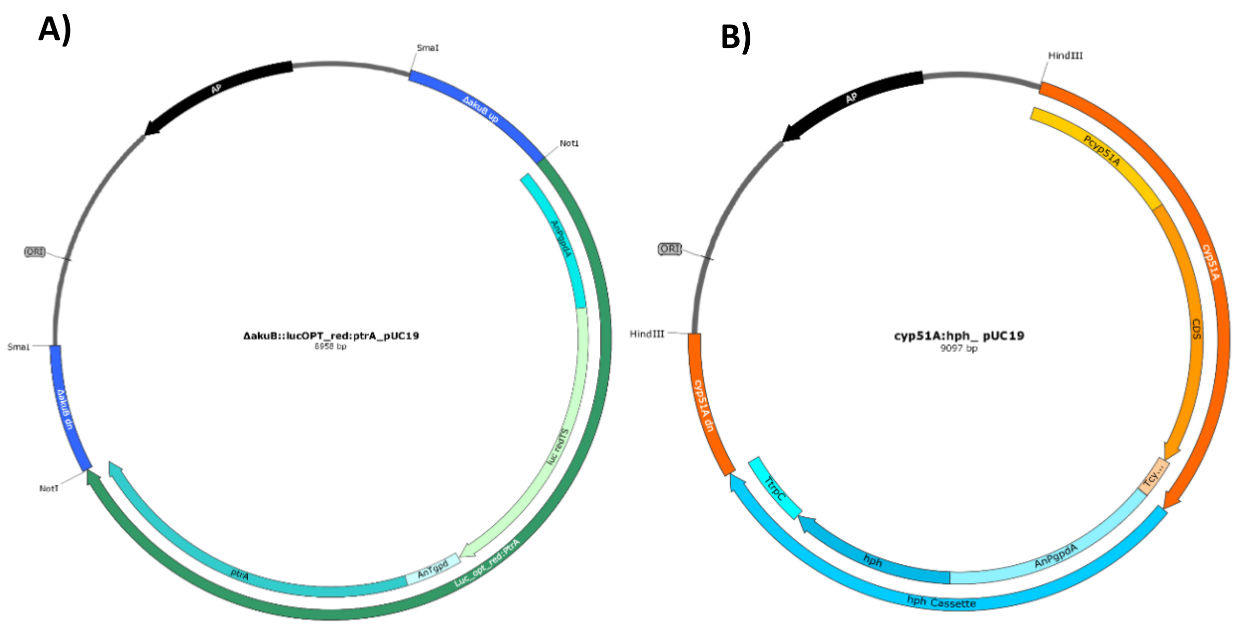

Fig. S1. Maps of plasmids generated in this study. A) Plasmid map of the *akuB* deletion cassette for expression of the red-shifted thermostable luciferase (*luc*_redTS) from the *akuB* locus in *A. fumigatus*. The cassette was excised by *Sma*I restriction and used for transformation of the *A. fumigatus* wild-type strain CBS144.89. **B)** Plasmid map of the cyp51A and hygromycin complementation cassette for the transformation of the genetically modified triazole-resistant *A. fumigatus* strains and reversion to the wild-type *cyp51A* gene. To perform the transformation of Af_Δ*akuB*::*luc*^{OPT_red}_ptrA_4003-new7 and Af_Δ*akuB*:: *luc*$_{OPT_red}$_ptrA_3216-1, the complementation construct was released from the vector backbone by *Hind*III restriction and gel-purified. **Abbreviations**: Upstream region of the *akuB gene (ΔakuB up),* glyceraldehyde-3-phosphate dehydrogenase gene *(gpdA)* promoter *(AnPgpdA)* and terminator *(AnTgpd)* region*, pyrithiamine resistance gene* (*ptrA*), downstream region *of the akuB* gene (*ΔakuB* dn), *cyp51A* gene (*cyp51A)* promoter (*Pcyp51A*), coding region (CDS) and terminator (T*cy*) regions, hygromycin B resistance gene (*hph*), tryptophan terminator (T*trpC*), downstream region of the *cyp51A* gene (*cyp51A* dn).

Disease Models & Mechanisms • Supplementary information

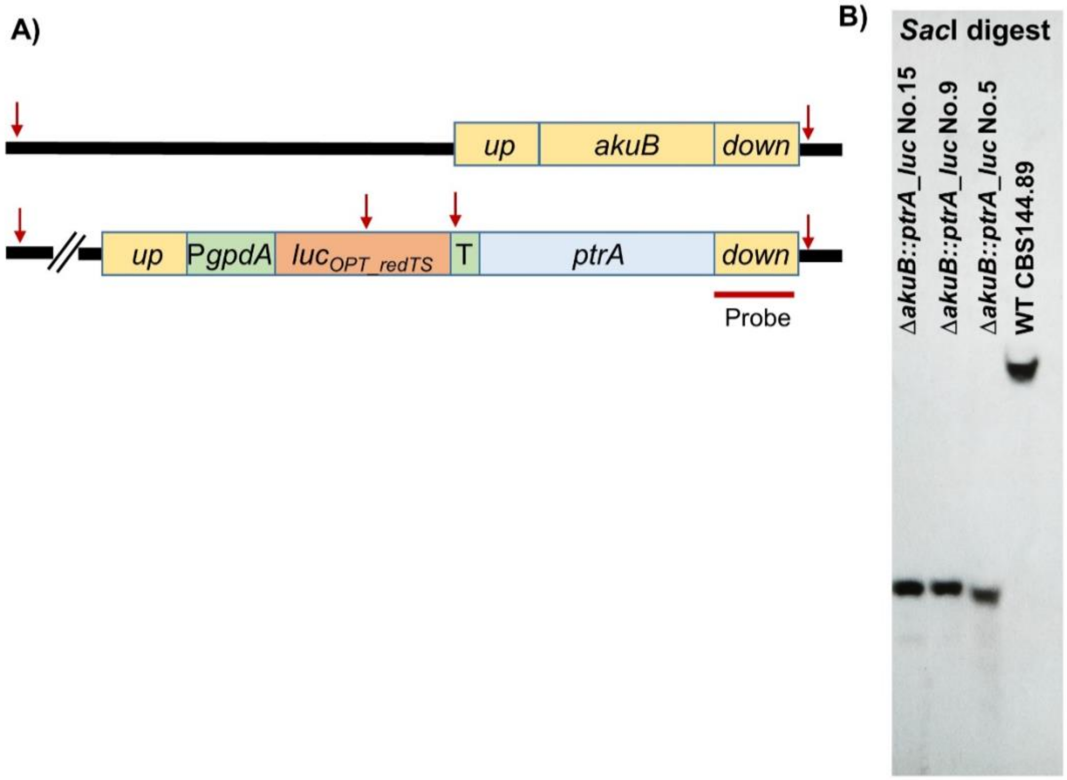

Fig. S2A. Southern blot analysis for confirmation of the replacement of the *akuB* locus in *A. fumigatus* by the luciferase expression construct. (A) Scheme of the genomic *akuB* locus in wild type and deletion mutants. The *akuB* locus was replaced by a codon-optimised and red-shifted thermostable firefly luciferase under control of the *gpdA* promoter (P*gpdA*) and its terminator (T). The pyrithiamine resistance gene (*ptrA*) served as marker in the transformation. *Sac*I restriction sites are indicated by arrows. A probe against the downstream region was generated. **(B)** Southern blot analysis of *Sac*I digested genomic DNA of wild type and deletion mutants showing the expected shift of the wild-type signal from 6193 bp to 3670 bp in the luciferase-expressing *akuB* deletion mutants. Strain No. 5 was used in subsequent experiments as parental strain.

Disease Models & Mechanisms • Supplementary information

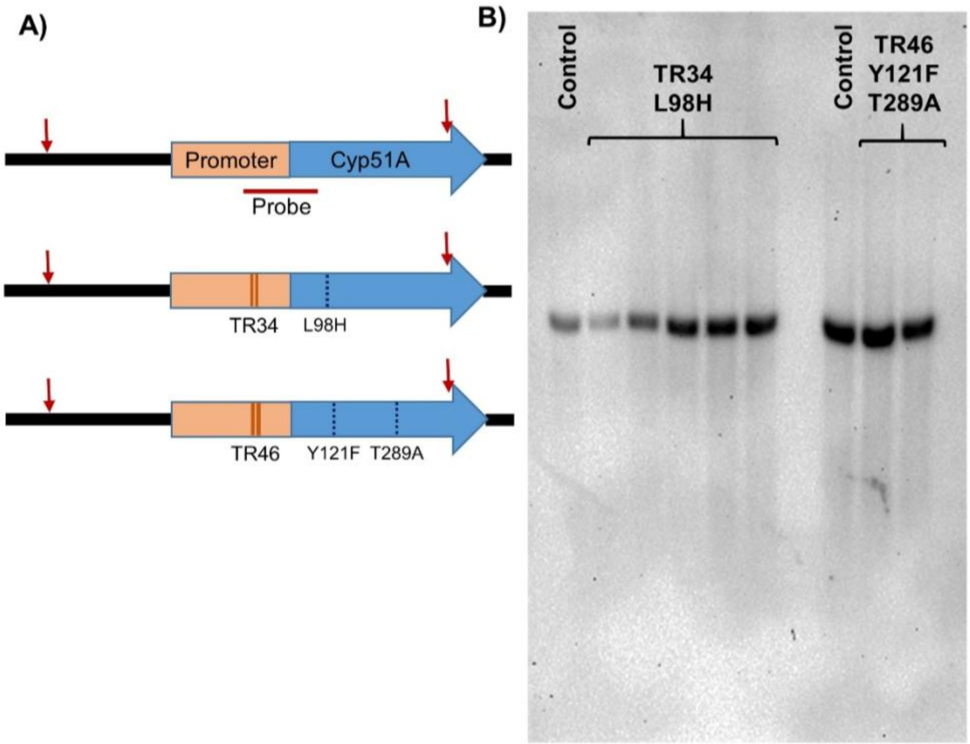

Fig. S2B. Southern blot analysis of itraconazole resistant *A. fumigatus* transformants. The parental Δ*akuB::luc* strain was transformed with the respective promoter and partial *cyp51A* gene fragment to achieve the desired mutations of the *cyp51A* gene. Transformants were regenerated on an itraconazole containing medium. **(A)** Scheme of the genomic situation of the *cyp51A* gene in the parental control strain and the *cyp51A* mutants. *Bam*HI restriction sites are indicated by arrows and result in a 3596 bp fragment for the *cyp51A* wild-type situation, 3630 bp for TR34/L98H mutants and 3642 bp for TR46/Y121/T289A mutants. **(B)** Southern blot analysis of *Bam*HI restricted genomic DNA of parental control strain and itraconazole resistant transformants. Only a single copy of the *cyp51A* gene is detected. Mutations in the *cyp51A* promoter and coding region were confirmed from selected strains by gene sequence analysis.

Disease Models & Mechanisms • Supplementary information

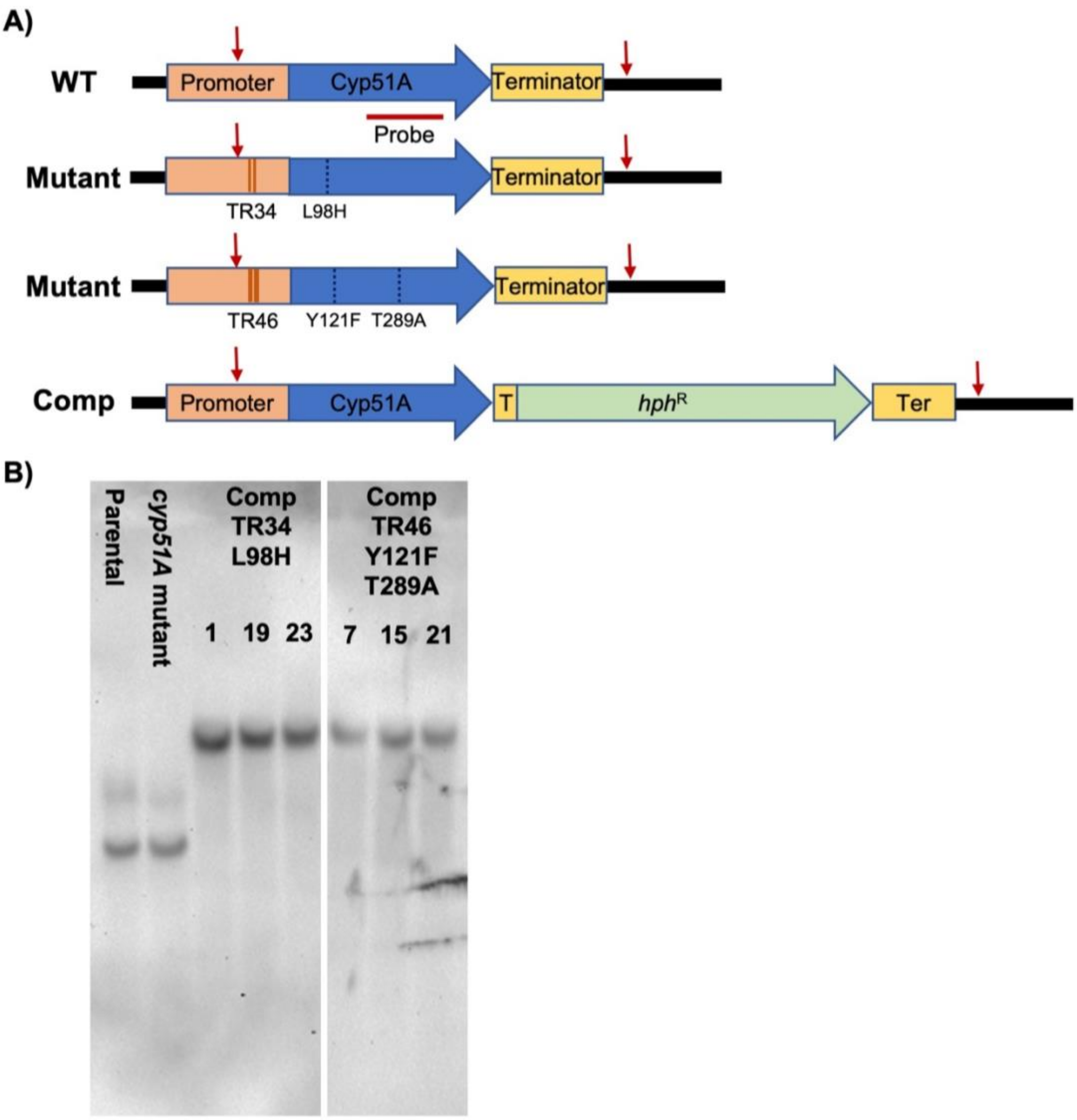

Fig. S2C. Southern blot analysis confirming the integration of the complementation construct into the *cyp51A* locus of triazole resistant mutants. The mutated *cyp51A* gene copy was replaced by the wild-type *cyp51A* gene using the hygromycin B resistance marker for selection of transformants. (A) Scheme of the genomic *cyp51A* gene situation in the in the parental control strain (WT), the *cyp51A* mutants and complemented mutants (Comp). *Xba*I restriction sites are indicated by arrows and result in a 3 kb fragment before and a 6 kb fragment after complementation. (B) Southern blot of *Xba*I restricted genomic DNA of parental control strain a *cyp51A* mutant strain and complemented mutants (Comp). A single signal with the expected size shift is observed in the complemented strains.

Disease Models & Mechanisms • Supplementary information

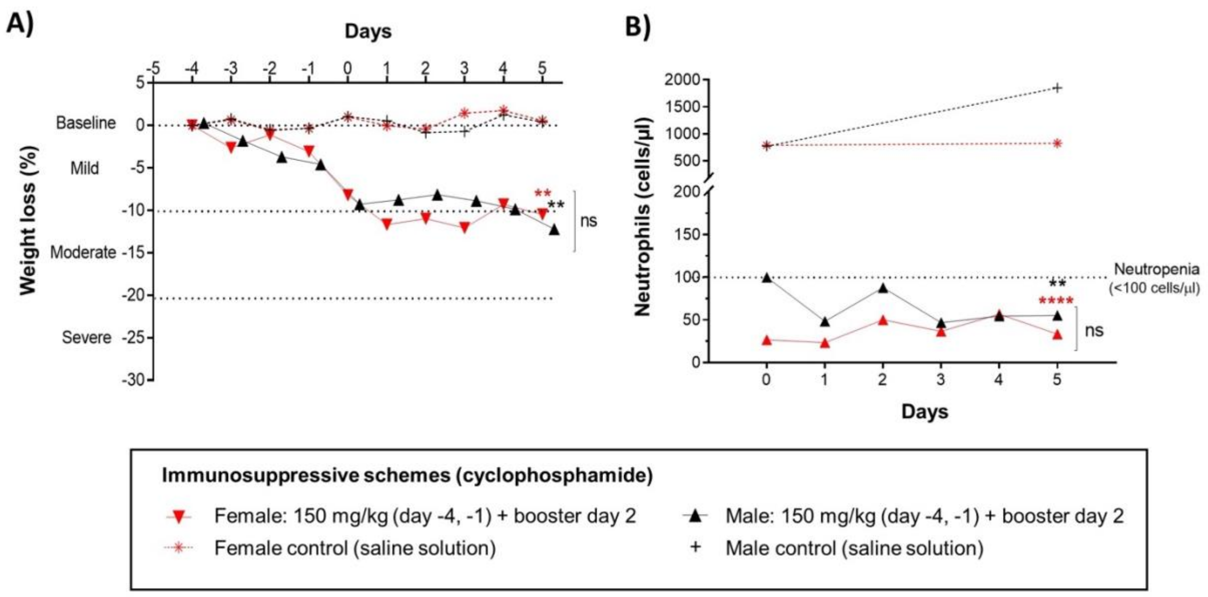

Fig. S3. Scheme of immunosuppressive effects on weight loss and neutropenia induction by cyclophosphamide injection with 150 mg/kg (day -4, -1) + booster at day 2 amongst male and female mice. A) Evolution of weight loss (% compared to baseline; mild 0-10%, moderate 10-20% and severe >20%) of tested cyclophosphamide immunosuppressive scheme between male and female mice and corresponding control group (saline solution) from day -4 (initiation of immunosuppressive therapy/baseline) until day 5. Graph represents mean value of weight loss percentage. **B)** Neutrophil count (cells/µl) kinetics after cyclophosphamide-induced immunosuppression between the female and male group and controls. Graph represents mean value (n = 3, per group per time point). *Mixed model ANOVA (multiple comparison analysis, Tukey correction)* to control group (above) and between gender *(lateral); * p = <0.05, ** p = <0.005, *** p = <0.0005, ns: non-significant.*

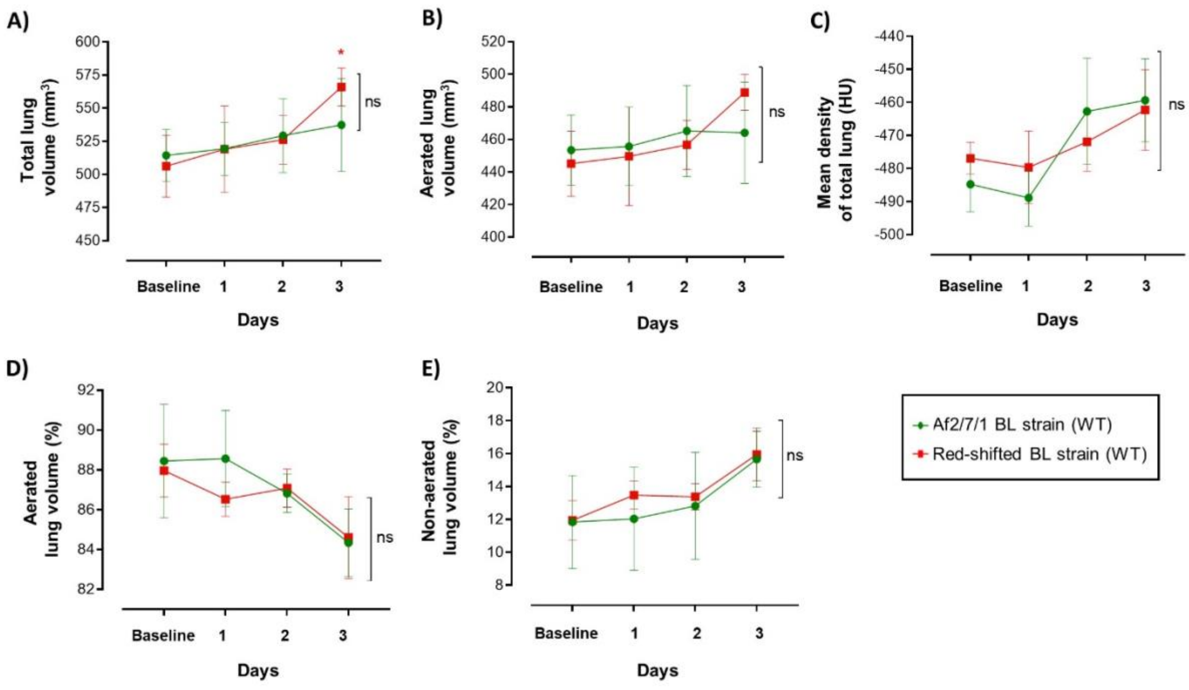

Fig. S4. Micro-CT-derived lung biomarkers of mice infected with the wild-type Af2/7/1 and Af_luc$_{OPT_red}$_WT ***Aspergillus fumigatus*** **strains.**

A) Micro-CT derived biomarkers quantifying the total lung volume and **B)** aerated lung volume of intranasally infected mice (5×10^5 spores) with the wild-type BL Af2/7/1/2 and Af_luc$_{OPT_red}$_WT strains. **C)** Mean density of total lung (HU) evolution over time. **D)** Aerated, and **E)** non-aerated lung volume percentages from total lung volume variances over time for infected mice. *Error bars represent SD of the results (mean) from multiple mice (n = 5 male). Two-way repeated measures ANOVA (multiple comparison analysis, Tukey correction); * p = 0.05, to baseline (above) and between groups (lateral).* ***Abbreviations:*** *Wild type (WT), Bioluminescent (BL), micro-computed tomography (micro-CT), Hounsfield Unit (HU), non-significant (ns).*

Disease Models & Mechanisms • Supplementary information

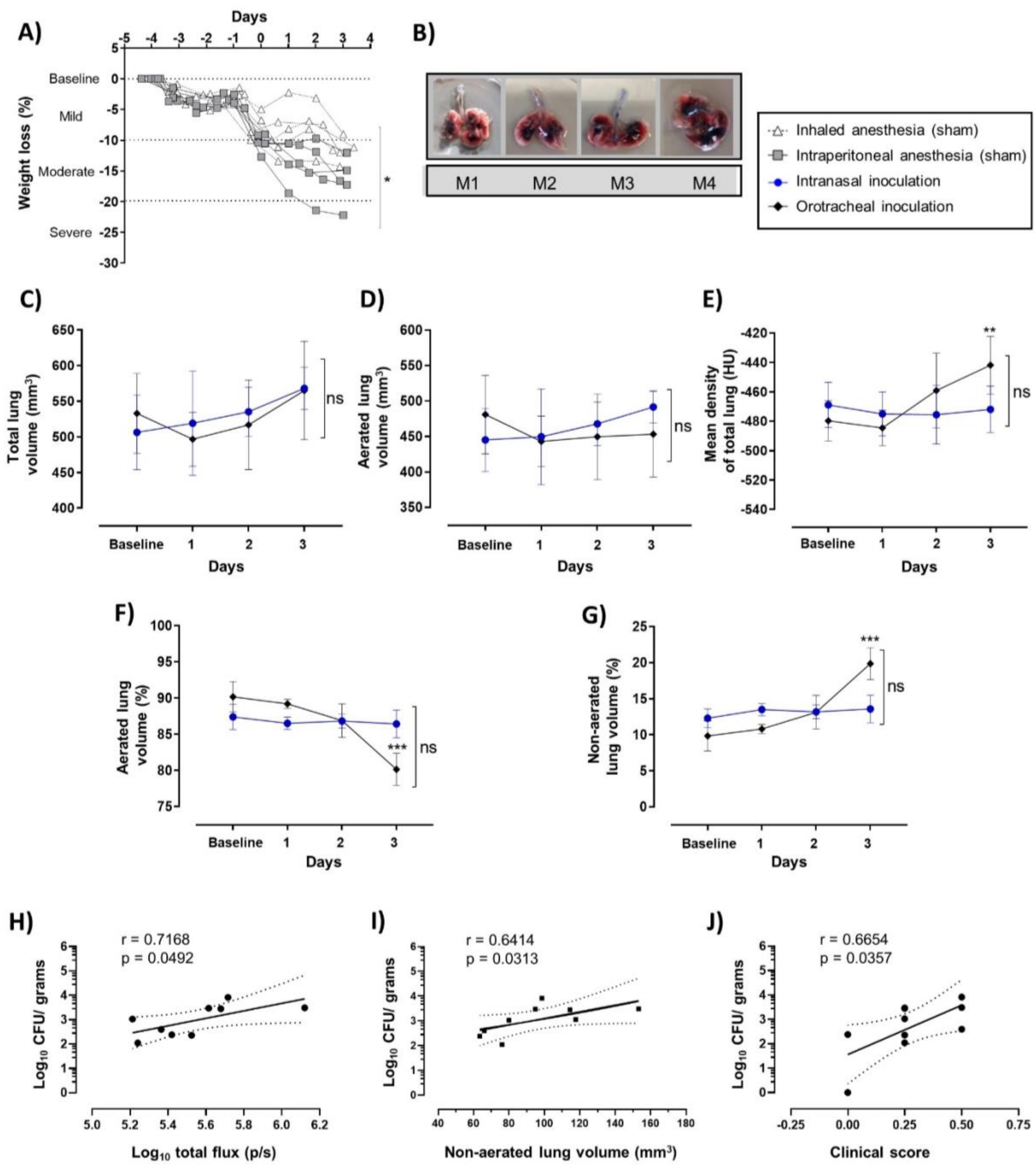

Fig. S5. Orotracheal route of inoculation characterization of non-infected mice, micro-CT derived biomarkers and correlation of imaging biomarkers from orotracheally and intranasally infected mice. A) Relative weight loss evolution in non-infected mice orotracheally sham-infected (25 µl saline-solution) immunocompromised mice after inhaled or peritoneally injected anaesthesia. **B)** Non-infectious *ex vivo* orotracheal route technique-assessment in mice (n = 4 male) after instillation with trypan blue (25 µl). **C)** Quantitative total and **D)** aerated lung tissue volume measurements of intranasally and orotracheally infected mice (5×10^5 Af_luc$_{OPT_red}$_WT spores) over time. **E)** Mean total lung density volume per mouse group over the time course of infection (HU). Percentage variances of **F)** aerated and **G)** non-aerated lung volumes from total lung volume before (baseline) and after infection.

Disease Models & Mechanisms • Supplementary information

Correlation and linear regression analysis of infected mice of quantified imaging biomarker readouts: **H)** bioluminescent signal (log_{10} total flux total), **I)** lung lesion development (non-aerated lung volume; mm^3) and **J)** cumulative visual clinical score of lung lesion based on imaging scans to lung CFU counts (log_{10}) on day 3. *Error bars represent SD of the results (mean) from multiple mice (n = 5 male), dashed lines represent the 95% confidence band. Two-way ANOVA (multiple comparison analysis, Tukey correction), Pearson correlation; * p = <0.05, ** p = <0.005, *** p = <0.0005, to baseline (above) and between groups (lateral).* ***Abbreviations:*** *Wild type (WT), micro-computed tomography (micro-CT), non-significant (ns), Hounsfield Unit (HU), colony forming units (CFU).*

Disease Models & Mechanisms • Supplementary information

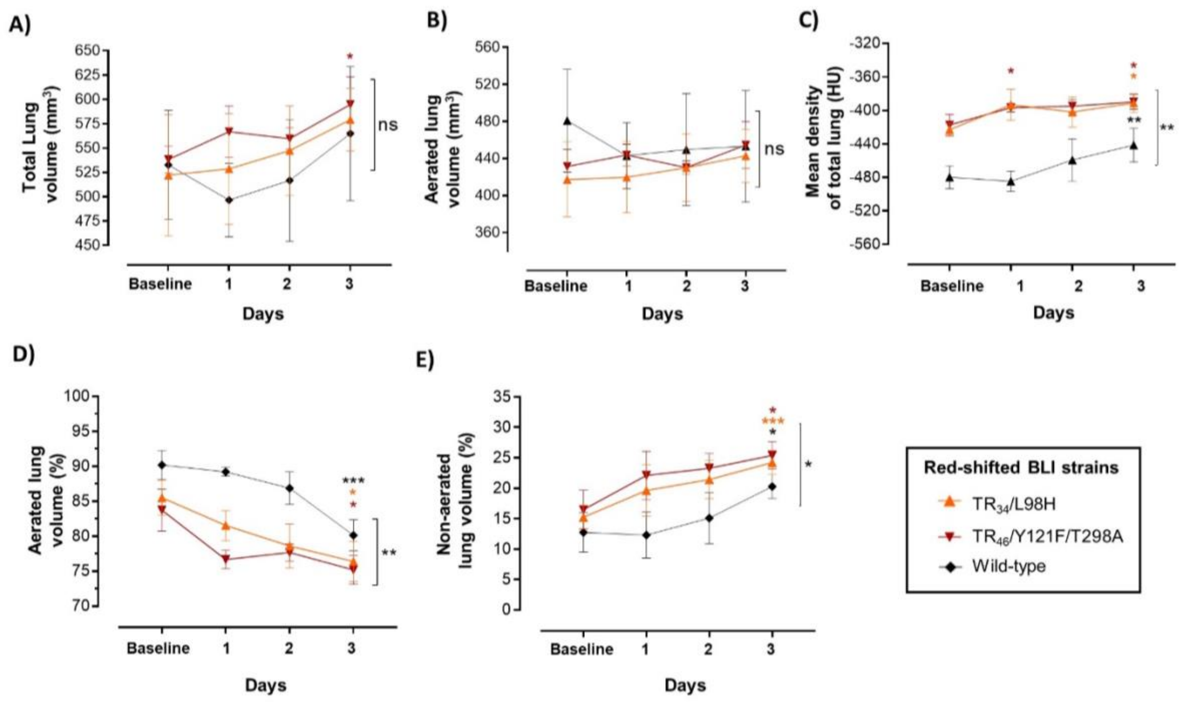

Fig. S6. Micro-CT derived lung biomarkers upon IPA development after infection with red-shifted luciferase expressing wild-type and triazole-resistant *Aspergillus fumigatus* strains.
A) Quantification of micro-CT derived total lung volume and **B)** aerated lung volume of mice orotracheally infected with red-shifted luciferase expressing and triazole-resistant (Af_luc$_{OPT_red}$_TR34, _TR46) and WT (Af_luc$_{OPT_red}$_WT) *A. fumigatus* strains (5×10^5 spores) before and after infection. **C)** Mean density of total lung volume over time (HU). **D)** Longitudinal aerated and **E)** non-aerated lung volumes percentages relative to total lung volume of infected animals upon infection per day. *Error bars represent SD of the results (mean) from multiple mice (n = 5 male). Two-way repeated measures ANOVA (multiple comparison analysis, Tukey correction); * p = <0.05, ** p = <0.005, *** p = <0.0005, to baseline (above) and WT (lateral).* ***Abbreviations:*** *IPA (invasive pulmonary aspergillosis), wild type (WT), micro-computed tomography (micro-CT), non-significant (ns).*

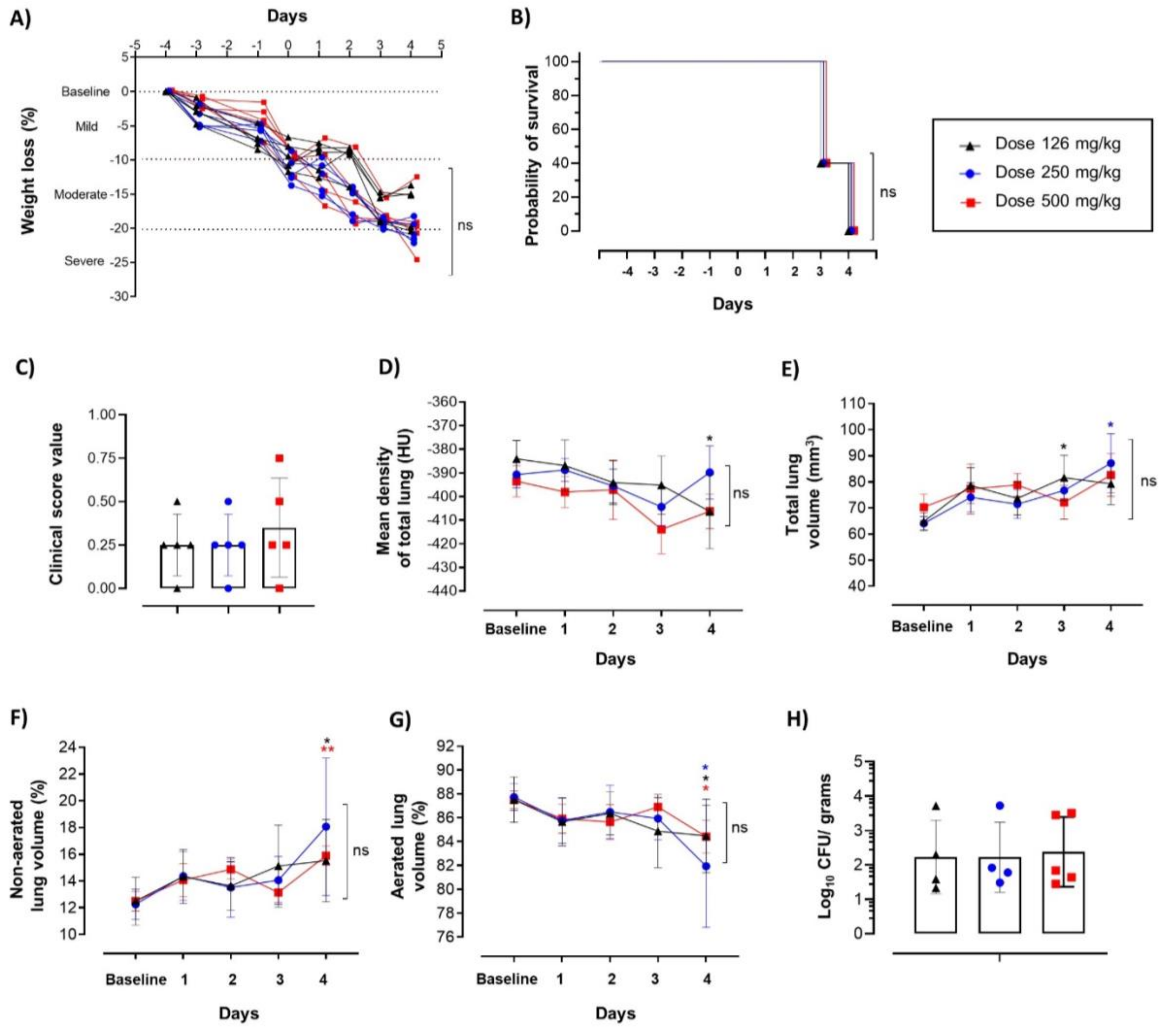

Fig. S7. Multimodal imaging of *in vivo* luciferin dose-dependent effects in *Aspergillus* infected mice A) Longitudinal weight loss percentage (%) evolution and **B)** survival comparison of orotracheally infected mice (Af_luc$_{OPT_red}$_WT 5 × 10^5 spores) according to the administered luciferin dose of 126 mg/kg, 250 mg/kg and 500 mg/kg. **C)** Micro-CT lung scan lesion development visual score (cumulative clinical score) of infected mice, grouped per luciferin dose. **D)** Longitudinal quantification of mouse lung micro-CT scans: mean density of total lung volume (HU) and **E)** non-aerated (lung lesion) lung volume (mm^3), grouped according to administered luciferin dose. **F)** Lung non-aerated and **G)** aerated lung volume percentage variances from total lung volume evolution over time, grouped per administered luciferin dose. **H)** Mean colony forming unit counts (log_{10} CFU/gram counts) of lung homogenates of infected mice per luciferin dose, evaluated at sacrifice (day 3-4 after inoculation). *Error bars represent SD of the results (mean) from multiple mice (n = 5 male). Two-way repeated measures ANOVA (multiple comparison analysis, Tukey correction; A, C-H), Log-rank (Mantel-Cox) test (B); * p = <0.05, ** p = <0.005; to baseline (above) and between groups (lateral).* ***Abbreviations:*** *Wild type (WT), micro-computed tomography (micro-CT), Hounsfield Units (HU), non-significant (ns), colony forming units (CFU).*

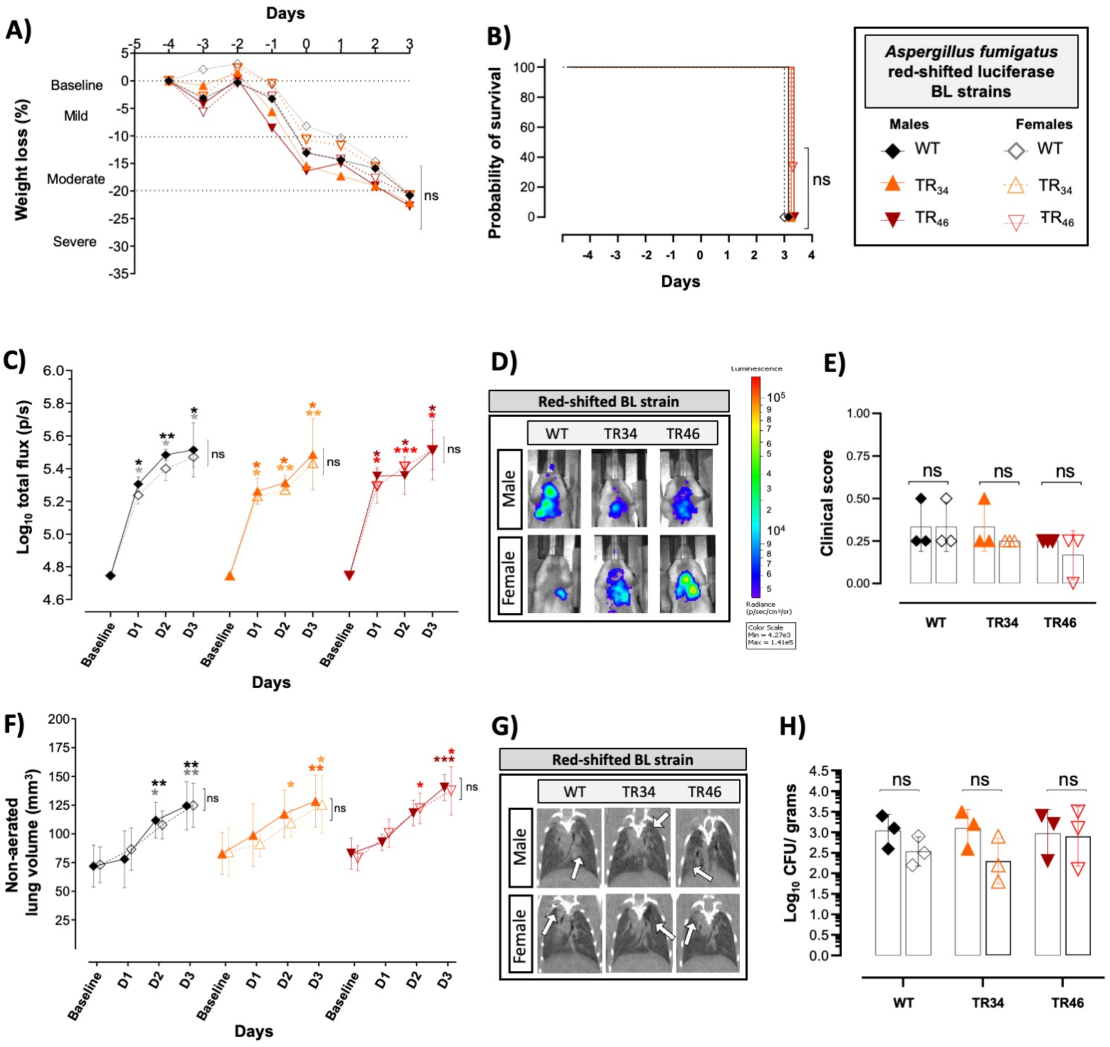

Fig. S8. Multimodal disease development comparison of bioluminescent *Aspergillus fumigatus* strains in male and female mice. A) Longitudinal weight loss percentage (%) and **B)** survival comparison of orotracheally inoculated cyclophosphamide immunosuppressed male and female mice with the Af_luc$_{OPT_red}$_WT, Af_luc$_{OPT_red}$_TR34 and Af_luc$_{OPT_red}$_TR46 strains (5×10^5 spores; n = 3 per group). **C)** Lung BL signals (log_{10} total flux) of infected mice before (baseline) and after infection, **D)** and representative BL images on day three after inoculation. **E)** Cumulative clinical scores representing visual assessment of lung lesion development on micro-CT scans and**, F)** quantitative evaluation of lung lesion development (non-aerated lung volume) over time. **G)** Representative coronal pulmonary micro-CT images of infected groups (arrows denote site of lesions). **H)** Colony-forming unit counts (log_{10} CFU/gram) from lung homogenates of infected mice at sacrifice (day 3 after inoculation). *Error bars represent SD of the results (mean) from multiple mice (n = 3), Two-way repeated measures ANOVA (multiple comparison analysis, Tukey correction; A,C,E,F,H), Log-rank (Mantel-Cox) test (B); * p = <0.05, ** p = <0.005, *** p = <0.0005.* ***Abbreviations:*** *Wild type (WT), bioluminescent (BL), micro-computed tomography (micro-CT), colony forming units (CFU), non-significant (ns).*

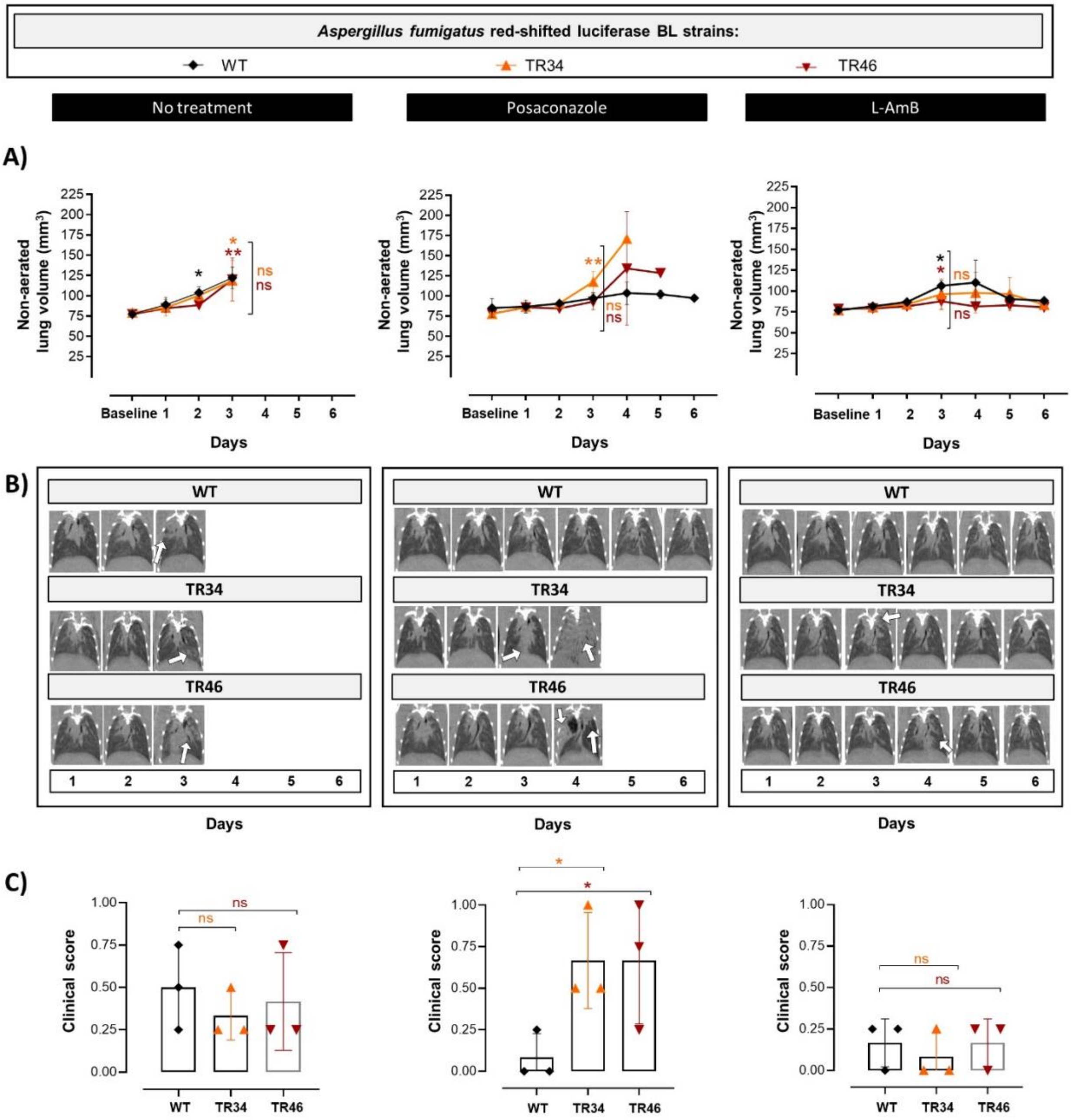

Fig. S9. Micro-CT biomarker comparison on IPA development after *in vivo* antifungal treatment of infected mice with triazole-susceptible and -resistant BL *A. fumigatus* strains. A) Lung lesion development (non-aerated lung volume) before (baseline) and after infection. **B)** Longitudinal representative coronal pulmonary micro-CT images of infected groups and **C)** cumulative clinical scores representing visual assessment of lung lesion development on micro-CT scans (arrows denote site of lesions). *Error bars represent SD of the results (mean) from multiple mice (n = 3 male). Two-way repeated measures ANOVA (multiple comparison analysis, Tukey correction); * p = <0.05, ** p = <0.005.* ***Abbreviations:*** *BL (bioluminescent), wild type (WT), micro-computed tomography (micro-CT), L-AmB (liposomal amphotericin B), non-significant (ns).*

Disease Models & Mechanisms • Supplementary information

Table S1. Blood cell analysis comparison of cyclophosphamide immunosuppressive schemes *in vivo*

Day	Blood analysis/ weight loss (%)	Cyclophosphamide 100 mg/kg (Day -4, -3, -2, -1, ± booster day 3)	Cyclophosphamide 100 mg/kg (Day -4, -3, -2, -1, + grapefruit juice)	Cyclophosphamide 150 mg/kg (Day -4, -1, ± booster day 2)		Cyclophosphamide 150 mg/kg (Day -4, -1, + grapefruit juice)	Control
**0**	White blood cells (cells/µl)	283.3	204.5	328.0		226.7	1656.7
	Neutrophils (cells/µl)	135.5	67.3	97.8		78.0	760.0
	Lymphocytes (cells/µl)	131.4	112.5	188.2		94.9	750.3
	Monocytes (cells/µl)	4.6	0.4	5.9		2.0	43.7
	Reticulocytes (cells/µl)	0.1	0.0	0.0		0.0	3.0
	Platelets (cells/µl x 10^3)	147.3	181.5	421.2		292.0	489.7
	Weight loss (%)	*10.1*	*17.3*	*9.6*		*15.8*	*+ 1.1*
**1**	White blood cells (cells/µl)	233.3	203.3	210.0		229.3	
	Neutrophils (cells/µl)	57.4	82.0	48.3		62.9	
	Lymphocytes (cells/µl)	150.7	65.8	145.6		138.4	
	Monocytes (cells/µl)	3.0	2.4	1.9		6.6	
	Reticulocytes (cells/µl)	0.0	0.0	0.1		0.1	
	Platelets (cells/µl x 10^3)	148.7	175.7	278.5		100.7	
	Weight loss (%)	*9.9*	*22.3*	*9.0*		*19.1*	*+ 0.1*
**2**	White blood cells (cells/µl)	243.3	583.3	278.3		185.0	
	Neutrophils (cells/µl)	102.8	110.73	87.7		109.9	
	Lymphocytes (cells/µl)	123.6	213.4	155.2		63.1	
	Monocytes (cells/µl)	8.4	11.2	13.6		3.3	
	Reticulocytes (cells/µl)	0.0	0.0	0.0		0.0	
	Platelets (cells/µl x 10^3)	189.7	179.8	282.0		132.3	
	Weight loss (%)	*7.8*	*25.0*	*8.4*		*20.9*	*+ 0.8*
				No booster	***Booster***		
**3**	White blood cells (cells/µl)	263.3	482.5	376.7	123.3	526.0	
	Neutrophils (cells/µl)	142.5	243	158.9	46.7	95.3	
	Lymphocytes (cells/µl)	109.4	341.1	195.5	35.5	350.2	
	Monocytes (cells/µl)	10.4	11.2	10.6	1.5	17.7	
	Reticulocytes (cells/µl)	0.0	0.0	0.0	0.0	0.2	
	Platelets (cells/µl x 10^3)	252.0	221.8	178.7	476.0	520.0	
	Weight loss (%)	*11.4*	*21.0*	*9.2*	*9.2*	*22.6*	*+ 0.7*

Disease Models & Mechanisms • Supplementary information

		No booster	***Booster***					
**4**	White blood cells (cells/µl)	350.0	540.0	2514.0	880.0	243.3	1804.0	1838.0
	Neutrophils (cells/µl)	101.1	132.1	900.3	118.4	54.4	650.9	413.8
	Lymphocytes (cells/µl)	206.2	344.0	1268.8	640.2	137.6	840.8	1275.7
	Monocytes (cells/µl)	30.1	16.4	93.2	32.5	0.5	55.2	34.9
	Reticulocytes (cells/µl)	0.0	0.1	1.1	0.1	0.0	0.7	2.6
	Platelets (cells/µl x 10^3)	228.3	329.0	472.6	571.7	502.3	329.2	501.0
	Weight loss (%)	*10.1*	*11.1*	*18.6*	*4.6*	*10.1*	*27.1*	+ *1.23*
**5**	White blood cells (cells/µl)		1881.7			128.3		
	Neutrophil (cells/µl)		544.1			55.2		
	Lymphocytes (cells/µl)		1127.3			57.8		
	Monocytes (cells/µl)		65.8			0.9		
	Reticulocytes (cells/µl)		1.5			0.0		
	Platelets (cells/µl x 10^3)		637.5			403.0		
	Weight loss (%)		*9.03*			*12.49*		

Grouped mean cell counts of 3 mice per analyzed regimen group per time point

Disease Models & Mechanisms • Supplementary information

Table S2. Primer sequences for the development of red-shifted luciferase expressing *Aspergillus fumigatus* bioluminescent strains

**Name**	**Sequence (5' → 3')**	**Function (Amplification)**
IF_Luc_PgpdA_f	ATG GAG GAC GCC AAG AAC ATC	Amplification of Af_*luc*$_{OPT\_red}$
IF_Luc_TgpdA_r	CTA GAC GGC GAT CTT GCCG	Amplification of Af_*luc*$_{OPT\_red}$
IF_PgpdAAn_f	CTG CCA ATT GGA TCC CGG GCT GAG TAA TAA GCG CAC	Amplification of *gpdA gene* promoter from *A. nidulans*
IF_PgpdAAn_Luc_r	CTT GGC GTC CTC CAT TGT GAT GTC TGC TCA AGCG	Fusion of *gpdA* gene promoter of *A. nidulans* with Af_*luc*$_{OPT\_red}$
IF_TgpdA_Luc_f	AAG ATC GCC GTC TAG GAA ACA GGT CGG AAG CC	Amplification of the terminator of *gpdA* gene of *A. nidulans*
IF_PgpdAter_r	GCG CTG CAG GGG CCC GGG TGG TAG CTC GTT GTC GAC	Amplification of the terminator of *gpdA* gene of *A. nidulans*
KU80upIFpUC_f	CGA GCT CGG TAC CCG GGA CTC AAT TTC TAT TCT AGA GCA TC	Amplification of upstream region of *akuB* and fusion with pUC19
KU80upIFPgpdA_r	GCG GCC GCC TCA GGG TCG TCA AAG TCA GTAC	Amplification of upstream region of *akuB* and fusion with *gpdA* gene promoter
PgpdAIFKu80up_f	CCC TGA GGC GGC CGC GGG CTG AGT AAT AAG	Amplification of P*gpdA*:Af_*luc*$_{OPT_red}$T*gpdA*_*ptrA* and fusion with upstream region of *akuB* gene
ptrAIFKU80do_r	AAC TTT GGG CGG CCG CGT ATT ATA CTG TC	Amplification of P*gpdA*:Af_*luc*$_{OPT_red}$T*gpdA*_*ptrA* and fusion with downstream region of *akuB* gene
KU80doIFptrA_f	GCG GCC GCC CAA AGT TAA AGG GCG CAA GC	Amplification of downstream region of *akuB* gene and fusion with *ptrA*
KU80doIFpUC_r	CTC TAG AGG ATC CCC GGG CTA TCA CTT TGC CCA GTC	Amplification of downstream region of *akuB* gene and fusion with pUC19
pCYP51A_f	CCT ATA AGT CGA GTG CAG GAC	Amplification of azole resistance gene (*cyp51A*)
tCYP51A_r	GAA GTC CTC GAT GGT TAC AAC	Amplification of azole resistance gene (*cyp51A*)
CypAfHind_up_f	CTG CAG GCA TGC AAG CTT CTT CAA TGC TCA GGC ATG	Amplification of the complementation *cyp51A* construct and fusion with pUC19
CypAfNotDown_f	*GCA CAG CGG CCG CAC GCA AAG ACG AGA AGG*	Amplification of the complementation *cyp51A* construct and fusion with pUC19
CypAfNotTer_r	GCG TGC GGC CGC TGT GCA CTG TTC TGG TTCC	Amplification of the complementation *cyp51A* construct and fusion with pUC19
CypAfHindDown_r	CAT GAT TAC GCC AAG CTT GAA CAT CGA ACC TCT CGT GTG	Amplification of the complementation *cyp51A* construct and fusion with pUC19

Disease Models & Mechanisms • Supplementary information

Table S3. *In vitro* mycological characteristics of genetically modified *Aspergillus fumigatus* strains

	**Parental Strain**	**Complemented strain**	**Parental Strain**	**Complemented strain**
	Af_luc$_{OPT_red}$_TR34	Af_luc$_{OPT_red}$_TR34 + *WTcyp51A -TR$_{34}$/L98H* (comp 3216-1, Δ*akuB*::*luc* No. 23 hygR)	Af_luc$_{OPT_red}$_TR46	Af_luc$_{OPT_red}$_TR46 +*WTcyp51A -TR$_{46}$/Y121F/T289A* (comp4003new7, Δ*akuB*:: *luc* No. 15 hygR)
Characteristics				
**Susceptibility (MIC; mg/L)** [1]				
Voriconazole	8	1	>16	0.5
Posaconazole	0.5	0.125	0.5	0.125
Itraconazole	16	0.5	16	0.25
Phenotype [2]	Resistant	WT	Resistant	WT
***Cyp51A* gene mutation**				
	TR$_{34}$/L98H	-	TR$_{46}$/Y121F/T289A	-

[1] EUCAST method for susceptibility testing of molds (version 9.3.2). [2] EUCAST clinical breakpoints version 10.0 (Resistance MIC values: itraconazole > 1, voriconazole > 1, posaconazole > 0.25 mg/L). ***Abbreviations:*** *MIC (minimum inhibitory concentration), WT (Wild type= susceptible to triazole antifungals).*

Disease Models & Mechanisms • Supplementary information
